# Metabolic reprogramming underlies metastatic potential in an obesity-responsive murine model of metastatic triple negative breast cancer

**DOI:** 10.1038/s41523-017-0027-5

**Published:** 2017-07-17

**Authors:** Ciara H. O’Flanagan, Emily L. Rossi, Shannon B. McDonell, Xuewen Chen, Yi-Hsuan Tsai, Joel S. Parker, Jerry Usary, Charles M. Perou, Stephen D. Hursting

**Affiliations:** 1 Department of Nutrition, University of North Carolina, Chapel Hill, NC 27517 USA; 2 Lineberger Comprehensive Cancer Center, University of North Carolina, Chapel Hill, NC 27517 USA; 3 Department of Genetics, University of North Carolina, Chapel Hill, NC 27517 USA; 4 Department of Pathology and Laboratory Medicine, University of North Carolina, Chapel Hill, NC 27517 USA; 5 Nutrition Research Institute, University of North Carolina, Kannapolis, NC 28081 USA

## Abstract

The vast majority of cancer-related deaths are due to metastatic disease, whereby primary tumor cells disseminate and colonize distal sites within the body. Triple negative breast cancer typically displays aberrant Wnt signaling, lacks effective targeted therapies, and compared with other breast cancer subtypes, is more likely to recur and metastasize. We developed a Wnt-driven lung metastasis model of triple negative breast cancer (metM-Wnt^lung^ **)** through serial passaging of our previously described, nonmetastatic, claudin-low M-Wnt cell line. metM-Wnt^lung^ cells displayed characteristics of epithelial-to-mesenchymal transition (e.g., increased invasiveness) with some re-epithealization (e.g., increased adhesion, tight colony formation, increased E-cadherin expression, and decreased Vimentin and Fibronectin expression). When orthotopically transplanted into syngeneic mice, metM-Wnt^lung^ cells readily formed tumors and metastasized in vivo, and tumor growth and metastasis were enhanced in obese mice compared with non-obese mice. Gene expression analysis revealed several genes and pathways altered in metM-Wnt^lung^ cells compared with M-Wnt cells, including multiple genes associated with epithelial-to-mesenchymal transition, energy metabolism and inflammation. Moreover, obesity caused significant transcriptomic changes, especially in metabolic pathways. Metabolic flux analyses showed greater metabolic plasticity, with heightened mitochondrial and glycolytic energetics in metM-Wnt^lung^ cells relative to M-Wnt cells. Similar metabolic profiles were found in a second triple negative breast cancer progression series, M6 and M6C cells. These findings suggest that metabolic reprogramming is a feature of metastatic potential in triple negative breast cancer. Thus, targeting metastases-associated metabolic perturbations may represent a novel strategy for reducing the burden of metastatic triple negative breast cancer, particularly in obese women.

## Triple negative breast cancer: Obesity and metabolism fuel disease spread

Metabolic changes contribute to the metastatic potential of triple negative breast cancer (TNBC), a mouse study shows. Stephen Hursting and colleagues from the University of North Carolina at Chapel Hill, USA, established metastatic mouse TNBC cells driven by Wnt-1, a signaling protein that’s highly active in this aggressive subtype of breast cancer. In a lab dish, these cells showed signs of increased invasiveness; and when transplanted into mice, the cells readily formed tumors that metastasized to the lungs. Obese mice experienced more aggressive tumor growth and spread than normal-weight animals. Gene expression analyses revealed that TNBC cells with metastatic potential have an energetic leg-up over their non-metastatic counterparts in the face of obesity-induced metabolic changes, suggesting that targeting metabolic perturbations could help reduce the burden of metastatic TNBC, particularly for obese women.

## Introduction

Metastasis is the dissemination and formation of secondary tumors at distal sites that causes most of the morbidity and mortality associated with triple-negative breast cancer (TNBC) and most other cancer types.^1, 2^ Key characteristics of metastatic cells include the ability to migrate, invade surrounding tissue, to survive in the periphery, and to attach to and colonize distal sites in the body. This progression involves epithelial cells within the primary tumor adopting mesenchymal properties, known as epithelial-to-mesenchymal transition (EMT), a key process in development, wound healing and stem cell biology.^3^ EMT is driven by signaling through Wnt, Notch, and TGFβ pathways and is mediated by several transcription factors including Snails, ZEBs, TWISTs and FOXOs, which control expression of genes involved in adhesion, migration, and invasion.^4^ The EMT program has been associated with the multistep cascade of the metastatic process,^5^ but the relevance of EMT to metastasis remains unclear. Mesenchymal tumor cells, having undergone EMT, share several ‘stemness’ characteristics of experimentally defined tumor-initiating cells (TICs), also referred to as cancer stem cells. Once cells reach a new organ, they often undergo mesenchymal-to-epithelial transition (MET), a re-differentiation program antithetical to EMT that may facilitate colonization and proliferation at the new site.^6^ Little is known about the dynamics of these programs in breast cancer, including the impact of obesity, due in part to a dearth of established preclinical models of progression.

The TNBC breast cancer subtype is characterized by the absence of expression of HER2, estrogen receptor and progesterone receptor and comprises 15–20% of all breast cancers in the US and includes intrinsic subtypes such as basal-like and claudin-low.^7–9^ TNBC is more likely to recur and metastasize than other subtypes, with 40% of patients presenting with positive lymph nodes at diagnosis.^10^ Unlike other subtypes, TNBC does not respond to hormone-targeted therapies and treatment is limited to cytotoxic chemotherapy.^11^ Furthermore, the obesity epidemic contributes to the burden of TNBC, as obesity is an established risk factor for development of the disease and may be associated with progression to metastasis.^12–14^ Emerging features of TNBC, and basal-like and claudin-low TNBC in particular, include aberrant Wnt/β-catenin signaling, stem cell-associated gene expression, and poor morphologic differentiation.^8, 15^

We previously established nonmetastic TNBC cell lines (E-Wnt and M-Wnt), derived from spontaneous tumors from the MMTV-Wnt1 transgenic mouse that, when orthotopically transplanted into syngeneic C57BL/6 mice, induce minimally invasive, nonmetastatic, epithelial-like mammary tumors or highly invasive mesenchymal tumors, respectively.^16^ Obesity enhances transplanted M-Wnt, but not E-Wnt, tumor growth. In the current study, we developed and characterized an obesity-responsive, metastatic model of TNBC derived from M-Wnt cells (hereafter referred to as ‘metM-Wnt^lung^ cells’**)** and identified a number of pathways altered during progression to metastasis, particularly metabolic alterations. Our findings highlight energy metabolism as a rational target in the treatment or prevention of metastatic TNBC.

## Results

### In vitro characterization of metM-Wnt^lung^ cells

**Fig. 1:**
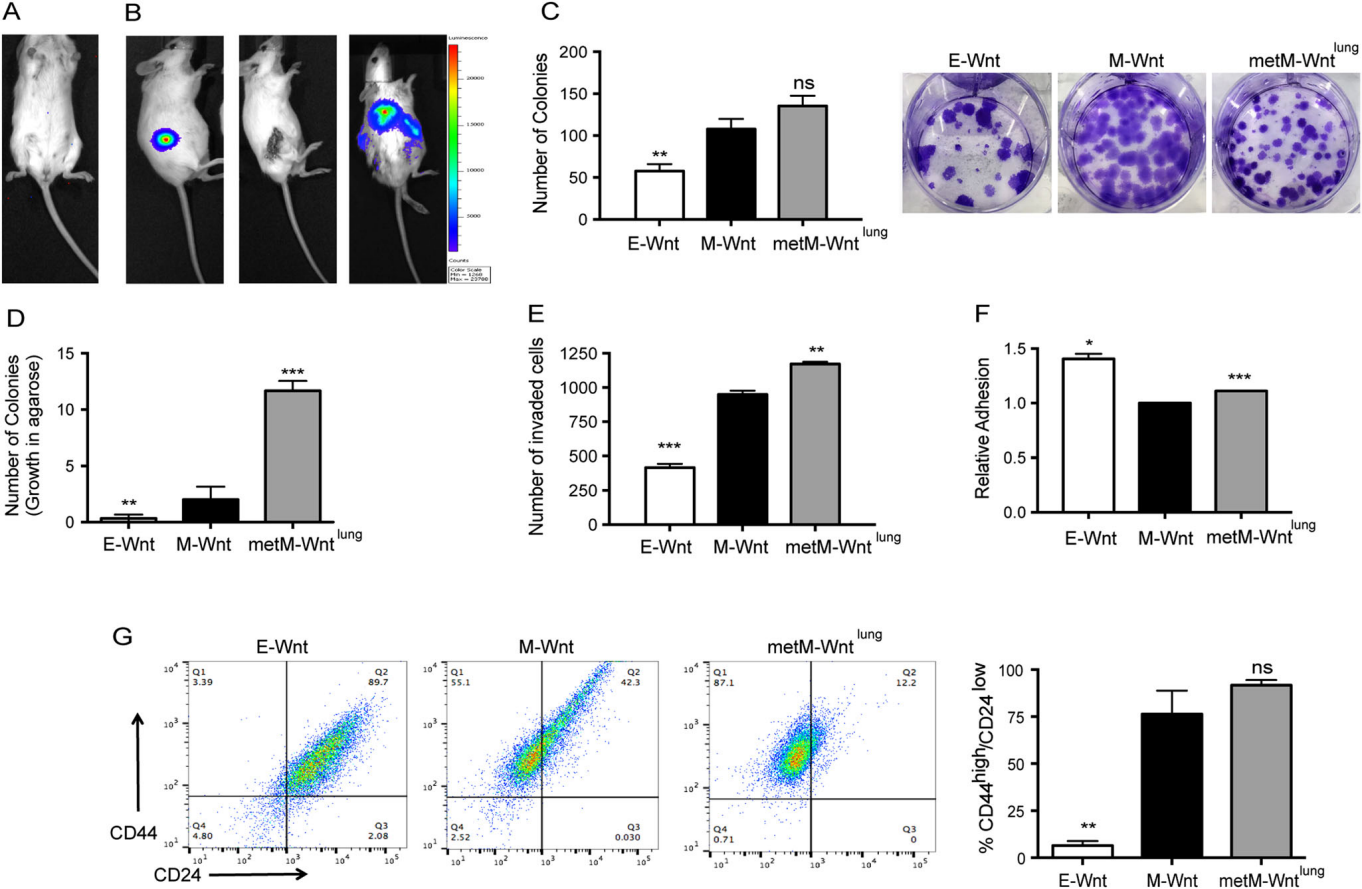
Generation and in vitro characterization of metM-Wnt^lung^ cells. **a** Representative IVIS image of C57BL/6 mice injected via tail vein with GFP-Luciferase expressing M-Wnt cells, showing no resulting metastasis. **b** Representative IVIS image of SCID mice injected with GFP-Luciferase expressing M-Wnt tumor brei, following survival surgery to remove tumor and resultant metastases in lung (left to right). **c** Colony formation assay of E-Wnt, M-Wnt, and metM-Wnt^lung^ cells and **d** colony formation in soft agarose. **e** Matrigel invasion assay and **f** adhesion assay of E-Wnt, M-Wnt and metM-Wnt^lung^ cells. **g** Representative flow cytometry analysis of E-Wnt, M-Wnt and metM-Wnt^lung^ cells stained with antibodies specific for CD44 (*y*-axis) and CD24 (*x*-axis), quantified in right-hand graph. All in vitro experiments are inclusive of three independent experiments. *p < 0.05, **p < 0.01, ***p < 0.001, ns non-significant, as compared with M-Wnt

The metM-Wnt^lung^ cell line was generated by serial transplantation of green fluorescence protein (GFP)-luciferase labeled nonmetastatic M-Wnt cell line (Fig. 1a) in five generations of severe-combined immunodeficient (SCID) mice (*n* = 3). Following transplantation into the 5th generation, a survival surgery was conducted to remove the primary tumor, confirmed by in vivo imaging system (IVIS) imaging (Fig. 1b). Mice were monitored for luciferase-positive lung metastases (Fig. 1b), and once detected, tumors were harvested, cells were sorted by flow cytometry according to GFP expression, and the metM-Wnt^lung^ cell line was established. M-Wnt and metM-Wnt^lung^ cells displayed similar in vitro colony-forming potential (Fig. 1c), though metM-Wnt^lung^ colonies were less diffuse relative to M-Wnt colonies and formed aggregated colonies with tight cell-cell junctions (Fig. 1b). metM-Wnt^lung^ cells displayed increased anchorage-independent clonogenic potential (Fig. 1d). metM-Wnt^lung^ cells also displayed enhanced adhesion and invasion compared with M-Wnt cells (Fig. 1e and f). metM-Wnt^lung^ cells had a high prevalence (91.7%) of CD44^high^/CD24^low^ cells, a cell-surface marker expression profile often linked to mammary TIC enrichment (Fig. 1g). M-Wnt cells had 76.3% CD44^high^/CD24^low^ enrichment, while E-Wnt cells had just 6.4% CD44^high^/CD24^low^. Nonmetastatic human breast cancer cell lines (e.g., MCF-7) have a lower prevalence of CD44^high^/CD24^low^ cells, while almost 100% of the metastatic cell line MDA-MB-231 are CD44^high^/CD24^low^ ^17^

### Tumorigenicity and metastatic potential of metM-Wnt^lung^ cells

**Fig. 2:**
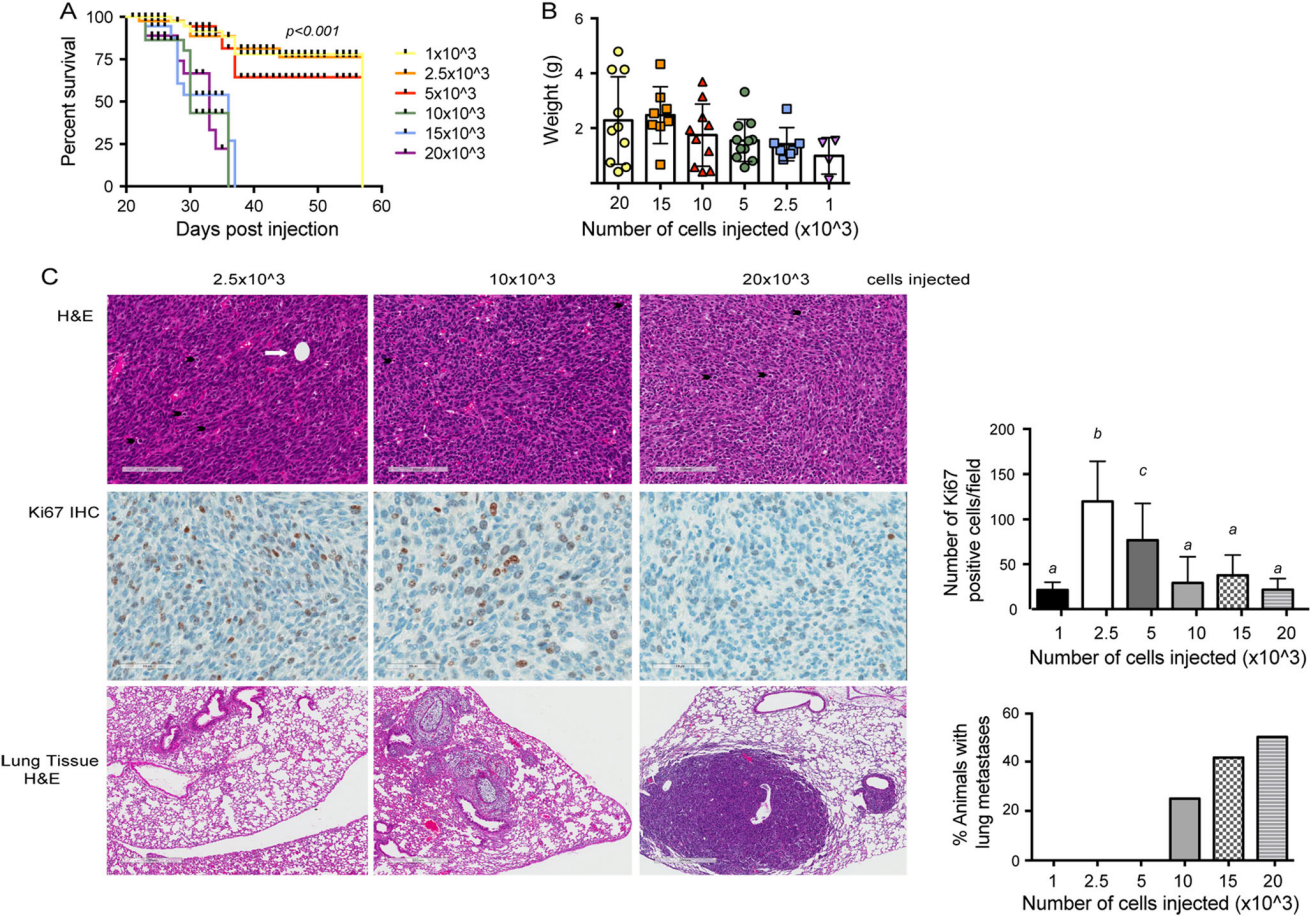
Tumorigenicity and metastatic potential of metM-Wnt^lung^ cells. **a** Survival curve (time until tumor reached 1.5 cm in any direction) of C57BL/6 mice transplanted with increasing concentrations of metM-Wnt^lung^ cells into the fourth mammary fat pad (1 × 10^3^, 2.5 × 10^3^, 5 × 10^3^, 10 × 10^3^ 15 × 10^3^, or 20 × 10^3^ cells per mouse; *n* = 12/group). **b** Resultant tumor weights of cells transplanted in (**a)**. **c** Representative micrographs of metM-Wnt^lung^ mammary tumors stained with H&E (top panel, scale bar 100 μm) and proliferative marker Ki67 (middle panel, 50 μm), and lung tissue stained with H&E (bottom panel 500 μm) from mice transplanted with the number of cells indicated. Bar graphs show the average number of proliferative cells per field (six fields chosen at random, *n* = 6 tumors) and incidence of metastatic lung tumors, mean ± SD. *White arrow* indicates intratumoral adipocyte, *black arrows* indicate apoptotic cells

Limiting dilution analysis was performed by orthotopically transplanting metM-Wnt^lung^ cells into the mammary fat pads of female albino C57BL/6 mice (1 × 10^3^, 2.5 × 10^3^, 5 × 10^3^, 10 × 10^3^, 15 × 10^3^, and 20 × 10^3^ cells per mouse; *n* = 12/group). Mammary tumors were palpable within 3 weeks in all six groups, with 100% of mice having tumors in the three higher doses in that time (data not shown). Survival (time until 1.5 cm tumor in any direction) was reduced with increasing cell dose (Fig. 2a), and resultant tumor weights were proportional to the dose of cells implanted (Fig. 2b). Histological analysis of metM-Wnt^lung^ tumors revealed a pleomorphic, poorly differentiated morphology, lacking ductal structures, and displaying regions of cell death, proliferative cells (Ki67 staining) and some intratumoral adipocytes (Fig. 2c), particularly in tumors arising from lower doses of cells.

Histological analysis of lung tissue collected from these animals confirmed the metastatic potential of metM-Wnt^lung^ cells, with metastatic lesions arising in the lungs in 50% of mice injected with 20 × 10^3^ cells/mouse; Fig. 2c, bottom panel. Metastatic tumors within the lung were pleomorphic, forming solid tumor nests and featuring areas of extensive fibrous stroma (Fig. 2c).

### Gene expression analysis of metastatic vs. nonmetastatic M-Wnt cells and comparison with human metastatic breast cancer

**Fig. 3:**
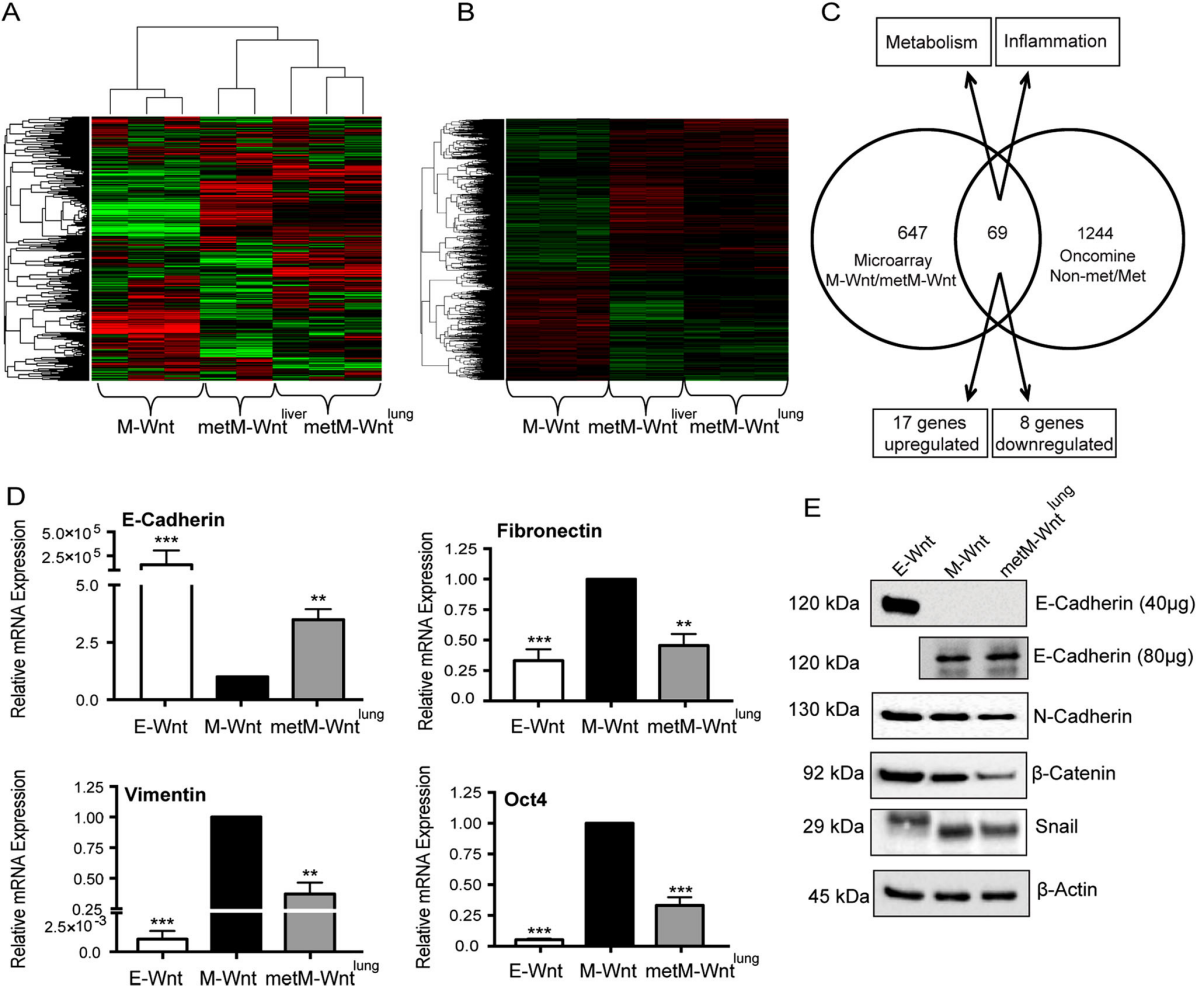
Gene expression analysis of metastatic vs. nonmetastatic M-Wnt cells and comparison with human metastatic breast cancer. **a** Heat map showing the top 5000 most variable probes in M-Wnt, metM-Wnt^lung^ and metM-Wnt^liver^ cells. **b** Heat map showing significantly different gene expression in metM-Wnt cell metM-Wnt^lung^) and metM-Wnt^liver^ cells (*q* < 0.001, FC > 3 or FC < 0.33) compared with nonmetastatic M-Wnt cells. **c** Venn diagram showing overlap between significantly different gene expression in metM-Wnt cells (*p* < 0.05, *q* < 0.001, FC > 3 or FC < 0.33) compared with nonmetastatic M-Wnt cells with comparisons of primary and metastatic breast cancer datasets in Oncomine (*p* < 0.001). **d** RNA expression of EMT-related genes in E-Wnt, M-Wnt and metM-Wnt^lung^ cells. Graphs show mean ± SD. *p < 0.05, **p < 0.01, ***p < 0.001, Student’s *t*-test. **e** Representative immunoblots showing protein expression of E-cadherin and N-cadherin, Snail and β-Catenin in E-Wnt, M-Wnt and metM-Wnt^lung^ cells, with β-Actin as a loading control

Gene expression microarray analysis revealed differential gene expression in metM-Wnt^lung^ cells compared with M-Wnt cells. The expression pattern in metM-Wnt^lung^ cells clustered tightly with a second, independent metastatic cell line derived (following same protocol as for metM-Wnt^lung^ cells) from a liver metastasis that arose from a transplanted M-Wnt mammary tumor (‘metM-Wnt^liver^ cells’) (Fig. 3a). We identified 721 genes (491 increased and 230 decreased *q* < 0.001 and FC > 3 or FC < 0.33)) commonly expressed in metM-Wnt^lung^ and metM-Wnt^liver^ metastatic cell lines and differentially expressed from parental, nonmetastatic M-Wnt cells (Fig. 3a; Supplementary File 1). Genes involved in EMT, inflammation, hypoxia and metabolic pathways were among the most significantly changed (Table 1).

**Table 1:** Pathway analysis of alterations in metastatic compared to non-metastatic M-Wnt cell lines

Pathway	# genes in set	*p*-value	Genes (fold difference metM-Wnt^lung^/M-Wnt)
Metabolism			
p53 Pathway	11	8.38E-08	*RGS16* (16.93); *RRAD* (7.2); *PROCR* (6.82); *HIST1H1A* (6.5); *PVT1* (5.83); *JAG2* (4.43); *PITPNC1* (3.79); *F2R* (3.43); *PTPRE* (3.36); *SLC7A11* (3.76); *PERP* (0.06)
Glycolysis	14	2.12E-07	*ANGPTL4* (44.23); *B3GNT3* (10.57); *ARTN* (8.85); *LHPP* (8.67); *NT5E* (7.08); *NOL3* (5.09); *CHST2* (4.79); *VCAN* (4.13); *LHX9* (3.83)*; TGFBI* (3.57)*; ENO2* (0.33); *RRAGD* (0.23)*; HS6ST2* (0.2); *AK3* (0.18)
Oxidative phosphorylation	2	8.27E-06	*PHYH* (7.23); *BDH2* (0.048)
MTORC1 signaling	2	2.35E-05	*SLC7A11* (3.76); *BCAT1* (3.25)
Upregulated by KRAS	25	0.000100599	*ITGBL1* (42.46); *IL2RG* (39.78); *TRAF1* (8.98); *MYCN* (8.39); *ID2* (8.48); *IRF8* (7.82); *TFPI* (7.13); *MMD* (5.79); *PLEK2* (5.6); *PLAT* (5.36); *PPBP* (5.22); *LAT2* (5.19); *PLAU* (5.01); *SEMA3B* (4.47); (4.23); *ADAM8* (4.85); *GPR124* (3.91); *IL33* (0.31); *CFHR2* (0.29); *GPNMB* (0.22); *EPB41L3* (0.1); *CFH* (0.1); *PRRX1* (0.05)
Xenobiotic metabolism	11	0.000237967	*PTGES* (24.65); *ID2* (8.48); *IRF8* (7.82); *IL1R2* (7.65); *NQO1* (7.32); *ALDH2* (4.07); *FBLN1* (3.92); *BCAT1* (3.25); ADH7 (3.21); *DDAH2* (0.28)*; ACOX2* (0.13)
Adipogenesis	11	0.000412562	*ANGPTL4* (44.23); *ANGPT2* (11.12); *OMD* (7.51); *LTC4S* (6.44); *SLC27A1* (5.6); *SORBS1* (4.17); *SNCG* (3.86); *REEP6* (3.19) *ALDH2* (4.07); *PHYH* (7.23); *EPHX2* (4.14)
Downregulated by KRAS	9	0.01248066	*CD80* (30.95); *EDN2* (23.5); *CAMK1D* (9.74); *PRODH* (7.29); *EDN1* (0.29); *SEPP1* (0.29); *YPEL1* (0.25); *MFSD6* (0.14)
Peroxisome	4	0.044372468	*CRABP2* (59.4); *HSD17B11* (10.78); *GSTK1* (5.7); *EPHX2* (4.14)
EMT			
Epthelial-to-mesenchymal transition	27	4.31E-08	*GLIPR1* (484.95); *PTHLH* (17.58); *IL15* (11.62); *TRAF1* (8.98); *ID2* (8.48); *COL6A3* (8.36); *MMP2* (7.85); *MYL9* (6.18); *COL3A1* (5.21); *ITGB3* (4.93); *VCAN* (4.13); *CRLF1* (4.03); *FBLN1* (3.92); *THBS1* (3.79); *COL12A1* (3.54); *TGFBI* (3.57)*; CTGF* (3.32); *SGCD* (3.41); *DAB2* (3.4); *ENO2* (0.33); *LOXL1* (0.31); *CDH11* (0.28); *MGP* (0.19); *TNFRSF11B* (0.14); *IL6* (0.14); *POSTN* (0.06); *PRRX1* (0.05)
Apical junction	12	0.000612388	*TRAF1* (8.98); *MMP2* (7.82); *MYL9* (6.18); *FYB* (4.16); *VCAN* (4.13); *AMIGO1* (3.79); *TGFBI* (3.57)*; DMP1* (3.55); *MYH9* (3.29); *ITGA10* (3.22); *CDH11* (0.28); *TNFRSF11B* (0.14)
Wnt/β-catenin signaling	2	9.02E-05	*JAG1* (5.79); *JAG2* (4.43)
Notch signaling	1	0.000643411	*JAG1* (5.79)
Apical surface	3	0.015807801	*IL2RG* (39.78); *HSPB1* (4.86); *RTN4RL1* (3.47)
TGFβ signaling	4	0.026356895	*BCAR3* (11.67); *ID2* (8.48); *LTBP2* (12.41); *THBS1* (3.79)
Hedgehog Signaling	3	0.044234222	*MYH9* (3.29); *NRCAM* (3.11); *NKX6-1* (3.06); *UNC5C* (0.18)
Inflammation			
Inflammatory response	24	1.35E-08	IFITM1 (58.48); *HRH1* (37.46); *PCDH7* (31.52); *RGS16* (16.93); *CXCR6* (16.15); *IL15* (11.62); *SEMA4D* (10.43) *AHR* (4.53); *CALCRL* (6.99); *CHST2* (4.79); *EDN1* (0.29); *F3* (3); *HAS2* (3.03); *IL18R1* (3.92); *IL1R1* (4.8); *IL6* (0.14); *ITGB3* (4.93); *LY6E* (4.08); *PIK3R5* (3.8); *PTGER4* (3.74); *PTPRE* (3.36); *SLC7A2* (0.25); *STAB1* (3.13); *TNFAIP6* (4.14);
TNFα/NFκB signaling	16	6.42E-07	*CD80* (30.95); *PHLDA2* (30.38); *TRAF1* (8.98); *ID2* (8.48); *EGR3* (7.59); *CXCR7* (6.39); *JAG1* (5.79) *PLAU* (5.01); *TNFAIP6* (4.14); *PTGER4* (3.74); *DUSP2* (3.37); *PTPRE* (3.36); *F3* (3); *SLC16A6* (0.32); *EDN1* (0.29); *IL6* (0.14)
Allograft rejection	13	4.73E-07	*IL2RG* (39.78); *CD80* (30.95); *IL15* (11.62); *IRF8* (7.82); *IL11* (4.57); *FYB* (4.16); *GCNT1* (3.93); *F2R* (3.43); *BCAT1* (3.25); *STAB1* (3.13); *IL7* (0.32); *ST8SIA4* (0.22); *IL6* (0.14)
IL-2/STAT5 signaling	16	2.15E-05	*ENPP1* (26.3); *RGS16* (16.93); *EOMES* (9.57); *TRAF1* (8.98); *IRF8* (7.82); *IL1R2* (7.65); *NT5E* (7.08); *TNFSF11* (6.65); *CKAP4* (4.92); *IL1RL1* (4.85); *AHR* (4.53); *IL18R1* (3.92); *PPAP2A* (3.36); *IFITM3* (3.05); *SPRY4* (0.31); *RRAGD* (0.23)*;*
IL-6/JAK/STAT3 signaling	10	0.004519815	*IL2RG* (39.78); *CD38* (10.86); *IL1R2* (7.65); *ITGB3* (4.93); *IL1R1* (4.8); *LEPR* (4.4); *IL18R1* (3.92); *PIK3R5* (3.8); *IL6* (0.14); *IL7* (0.32)
Interferon γ response	15	0.011933194	*P2RY14* (25.26); *EPSTI1* (12.98); *IL15* (11.62); *CD38* (10.86); *IRF8* (7.82); *ITGB7* (4.82); *EIF4E3* (4.5); *TNFAIP6* (4.14); *LY6E* (4.08); *APOL6* (3.57); *IFITM3* (3.05); *IL7* (0.32); *ST8SIA4* (0.22); *IL6* (0.14); *CFH* (0.1)
Coagulation	11	0.018260394	*CRIP2* (11.19); *MMP2* (7.85); *PLAT* (5.36); *PLAU* (5.01); *F3* (3); *ITGB3* (4.93); *THBS1* (3.79); *USP11* (0.29); *CTSH* (0.26); *ACOX2* (0.13); *CFH* (0.1)
Stress response			
Unfolded protein response	1	2.51E-07	*ERN1* (3.21)
UV response	6	4.70E-07	*RRAD* (7.2); *OLFM1* (3.65); *ENO2* (0.33); *GGH* (0.25); *CHKA* (0.21); *IL6* (0.14)
Apoptosis	12	6.56E-05	*HGF* (20.52); *IGFBP6* (17.67); *CD38* (10.86); *MMP2* (7.85); *EGR3* (7.59); *PLAT* (5.36); *HSPB1* (4.86); *F2R* (3.43); *IFITM3* (3.05); *ENO2* (0.33); *IL6* (0.14); *PMAIP1* (0.12)
Other			
Androgen response	4	2.88E-05	*INPP4B* (58.08); *MAF* (5.32); *PPAP2A* (3.36); *SEPP1* (0.29)
Angiogenesis	5	0.001119122	*JAG1* (5.79); *COL3A1* (5.21); *JAG2* (4.43); *VCAN* (4.13); *POSTN* (0.06)

Gene sets were identified using a permutation-based approach to test for enrichment across the univariate statistics of all genes in a set. The count of genes in each set that also surpass univariate criteria (*q* < 0.001 and FC > 3 or FC < 0.33) are listed. Altered genes were functionally clustered using the Hallmark gene sets in Molecular Signature Database (MSigDB) with a threshold set to *p* < 0.05. Number of genes reflects number of overlap from significant genes identified from this microarray analysis and those featured in Hallmark gene sets. Fold change in metM-Wnt^lung^ compared to M-Wnt are shown in parentheses in the right hand column

The shared gene expression signature between metM-Wnt^lung^ and metM-Wnt^liver^ cells, relative to M-Wnt cells, represents genes underlying metastatic potential of Wnt-driven TNBC. Comparing the 721 genes differentially expressed between parental and metastatic M-Wnt lines with available human breast cancer microarray datasets^18–21^ (The Cancer Genome Atlas; Gene Expression Omnibus expO), we discovered 69 commonly altered genes between human and mouse primary and metastastic lesions (Fig. 3a). Twenty five of these genes displayed fold changes of similar direction (17 genes overexpressed and 8 genes underexpressed) in both analyses (Table 2).

**Table 2:** List of genes concordant with available human breast cancer microarray datasets

Gene	Fold change metM-Wnt/M-Wnt	Fold change Oncomine Met/Nonmet
*ARHGAP8*	3.03	2.84
*BCAT1*	3.25	3.09
*ERGIC1*	3.75	1.42
*ESYT2*	3.42	2.9
*FLRT3*	5.11	6.37
*GALNTL2*	0.25	0.18
*GCNT1*	3.93	3.83
*HOXB13*	0.29	0.24
*HS3ST3A1*	4.3	4.7
*HSD17B1*	0.32	0.56
*HSPB1*	4.86	1.56
*IL6*	0.14	0.67
*MCTP1*	0.26	0.56
*MEGF10*	0.04	0.42
*MURC*	4.83	2.22
*NKAIN2*	0.04	0.63
*RALGAPA2*	0.24	0.2
*RALGPS2*	3.33	1.28
SEC16B	7.12	1.47
*SEMA4D*	10.43	2.34
*SH3GL3*	8.18	1.56
*SIGIRR*	48.42	1.78
*TAF9B*	27.1	1.27
*TRAPPC9*	3.9	1.5
*TSPAN12*	0.02	0.87

Genes were considered significant if *q* < 0.001 (metM-Wnt/M-Wnt) or *p* < 0.001 (Oncomine Met/Nonmet)

### Expression of EMT-related genes in metM-Wnt^lung^ cells

We next examined the expression of a panel of EMT-related genes in met-MWnt^lung^ cells. MetM-Wnt^lung^ cells displayed significantly increased mRNA expression of E-Cadherin (Fig. 3b), as well as significantly decreased mRNA of Fibronectin, Vimentin, and Oct4. Expression of TGF-β, FOXC2, Slug and Twist was unchanged (data not shown). Increased E-Cadherin expression was also observed at the protein level in metMWnt^lung^ cells, coincident with decreased N-Cadherin, β-Catenin and Snail expression (Fig. 3c). Snail was detected at a higher molecular weight in E-Wnt than both M-Wnt and metM-Wnt^lung^ lines, which may represent phosphorylated and unstable Snail protein.^22^ Taken together these results suggest that MET has occurred in metM-Wnt^lung^ cells, though they still retain some mesenchymal characteristics.

### Effects of obese phenotype on systemic metabolism and metM-Wnt^lung^ tumor growth and metastasis

**Fig. 4:**
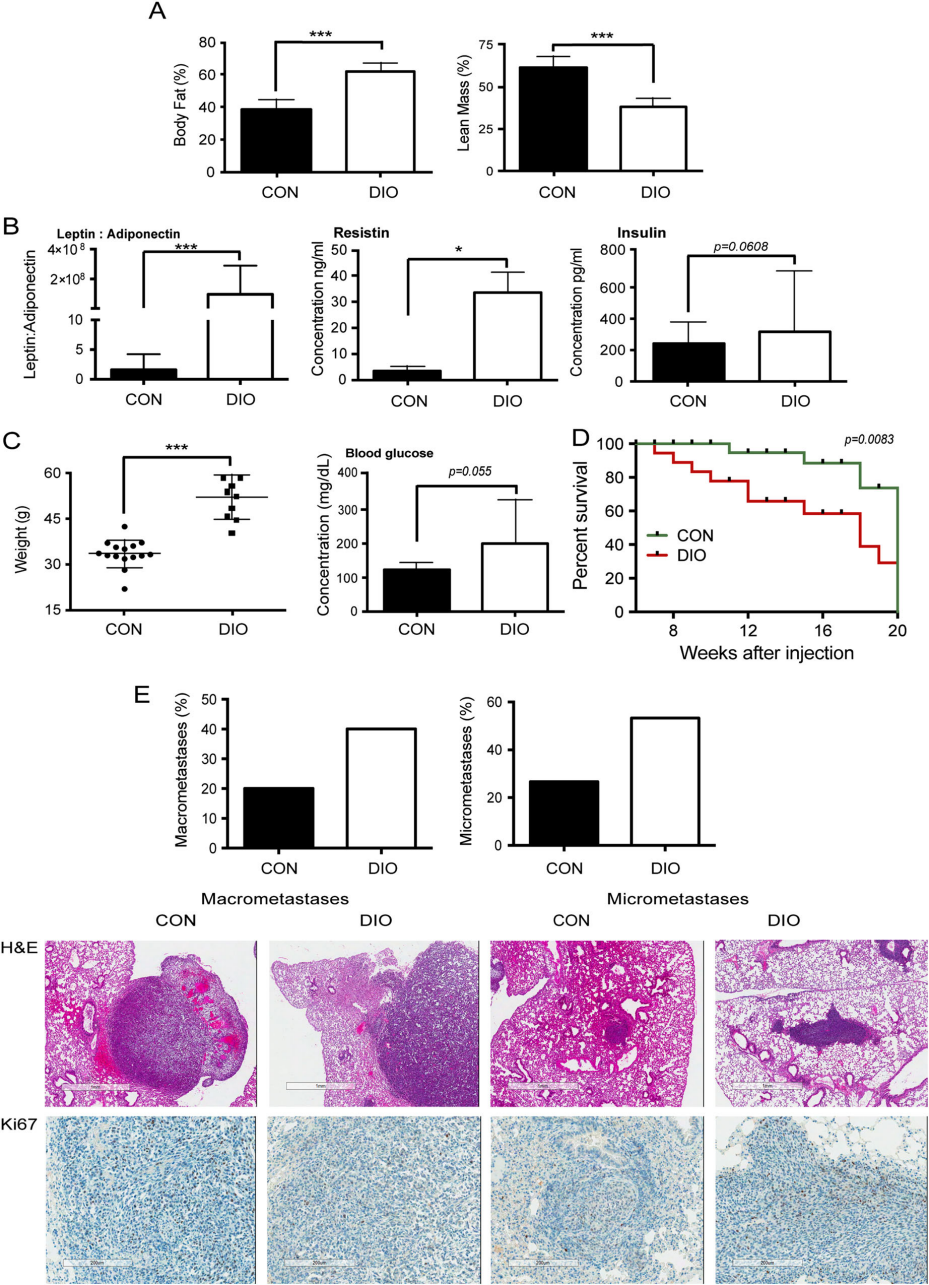
Effects of obese phenotype on systemic metabolism and metM-Wnt^lung^ tumor growth and metastasis. **a** Effects of diet-induced obesity (DIO) diet, relative to control (CON) diet, on body fat (i) and lean mass (ii) (*n* = 5/group). **b** Effects of DIO diet on metabolic hormones. (i) Serum leptin to adiponectin ratio and serum concentration of resistin (ii) and insulin (iii) (*n* = 5/group). **c** Body weight (i) and non-fasting blood glucose level (ii) of mice fed a control (*n* = 15) or DIO diet (*n* = 13). **d** Survival curve of mice fed a control or DIO diet after injection with 2.5 × 10^3^ metM-Wnt^lung^ cells in 200 μl into the tail vein. Mice were euthanized upon signs of any distress. *p* = 0.083, Gehan-Breslow–Wilcoxon test. **e** Extent of macrometastases and micrometastases in mice from survival study (Fig. 4D), as determined by gross pathology and histopathology, respectively. Bar graphs show the percentage of animals displaying macrometastases (control *n* = 3/15; DIO *n* = 5/13) and micrometastases (control *n* = 4/15; DIO *n* = 7/13). Representative micrographs of H&E (top panel) or Ki67 (bottom panel) stained serial sections (4 μm apart) of lung tissues from mice fed a control or DIO diet, showing metM-Wnt^lung^ macrometastases (left panels) and micrometastases (right panels). Bar graphs show the mean ± SD, *p < 0.05, ***p < 0.001, Student’s *t*-test

To model the postmenopausal state in non-obese vs. obese mice, 40 female ovariectomized albino C57BL/6 mice (8–10 weeks old) were placed on a control, low fat diet (10% kcal from fat), or a high fat diet-induced obesity (DIO; 60 kcal% fat) for 13 weeks. Five randomly-selected mice per group were then euthanized, serum collected for hormone analysis, and carcasses analyzed for body composition analysis (Fig. 4a and b). DIO, relative to control diet, resulted in a significant increase in body fat and reduction in lean mass (Fig. 4a). Increased systemic markers of metabolic dysregulation,^23^ including serum resistin (*p* < 0.05), the ratio of leptin to adiponectin (*p* < 0.001), and insulin (*p* = 0.061) (Fig. 4b) were found in DIO mice compared to control mice.

Remaining mice (*n* = 15/group) received a tail vein injection of metM-Wnt ^lung^ cells (2.5 × 10^3^ cells/mouse), remained on their diets and were monitored for signs of moribundity for the remainder of the study. Once mice began to exhibit signs of distress, they were weighed and blood glucose measured before euthanasia. Mice fed a DIO diet weighed significantly more at time of euthanasia compared with control mice (Fig. 4c), and also had increased non-fasting blood glucose (*p* = 0.055, Fig. 4c). DIO mice, relative to control mice, had reduced overall survival (Fig. 4c), a higher proportion of visible lung macrometastases (40% compared with 20%, Fig. 4e), and a higher incidence of lung micrometastases (53.8% vs. 26.7%, Fig. 4e). No significant between-group differences were detected in size or number of micrometastases (data not shown).

### Gene expression analysis in M-Wnt and metM-Wnt^lung^ cells in an in vitro model of obesity

To model obese vs. non-obese conditions in vitro (17), metM-Wnt^lung^ cells were cultured in media containing sera from DIO or control mice (replacing fetal bovine serum (FBS) in the culture medium). Gene expression microarray analysis revealed a signature of 98 genes potentially altered in both cell lines in the modeled obese vs. non-obese conditions (*p* < 0.001). Supplemental Files 2 and 3). Enrichment analysis was performed to minimize false discoveries, and highlighted genes included those involved in fatty acid metabolism, olfactory transduction, mTOR signaling and autophagy (Supplemental File 3).

### Metabolic alterations in Wnt cells differing in EMT and metastatic potential

**Fig. 5:**
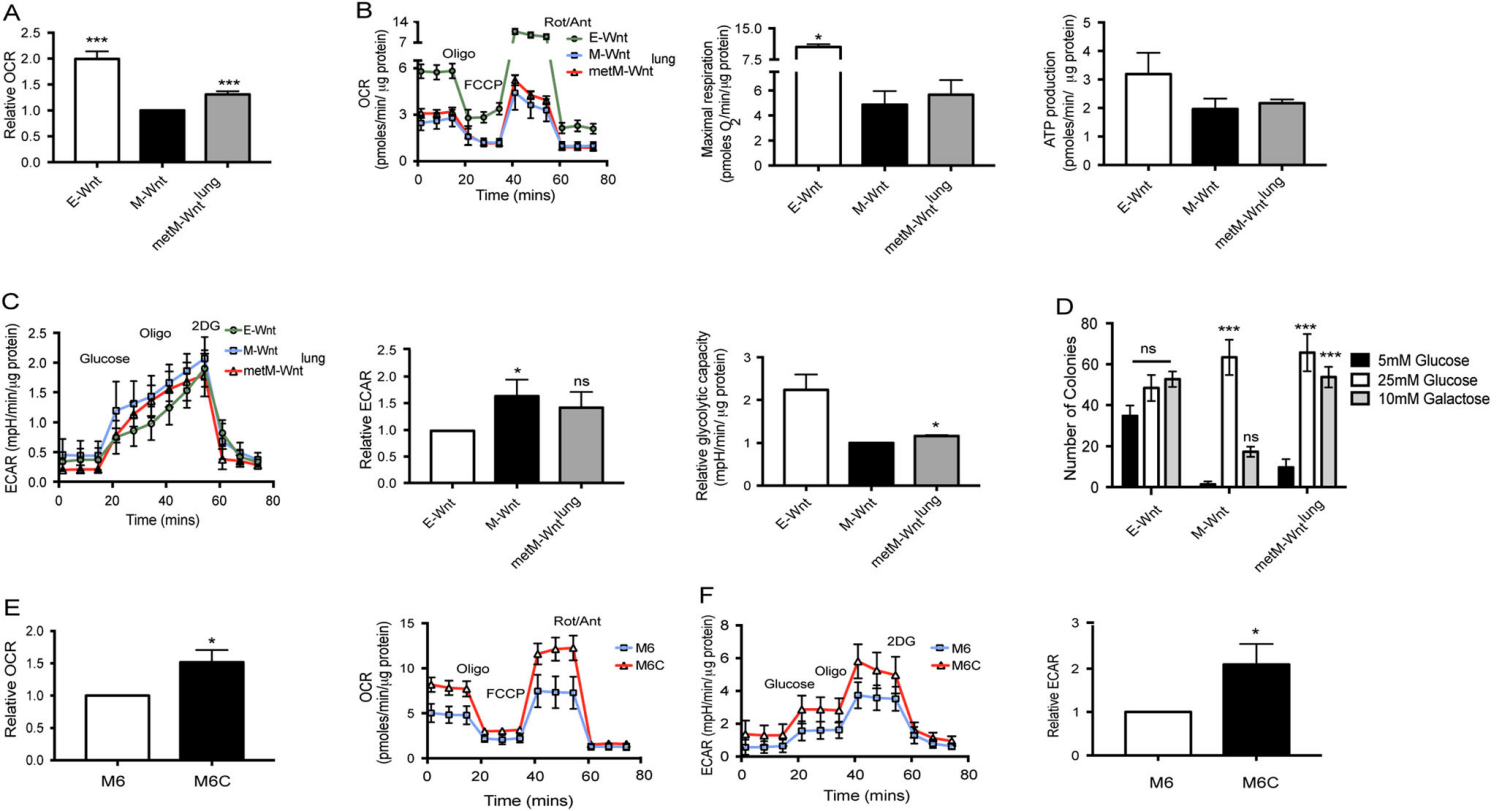
Metabolic alterations in metastatic compared to nonmetastatic TNBC cells. **a** Relative basal oxidative consumption rate (OCR) of E-Wnt, M-Wnt, metM-Wnt^lung^, as compared to M-Wnt cells. **b** Representative mitochondrial stress test of E-Wnt, M-Wnt, metM-Wnt^lung^ cells. Cells were injected with 1 μM oligomycin (oligo), followed by 1 μM carbonyl-cyanide-4-(trifluoromethoxy)phenyhydrazone (FCCP) and finally a combination of rotenone/antimycin (3 μM), (Rot/Ant). Uncoupling of the electron transport chain (ETC) from ATP synthesis (FCCP) reveals maximum oxidative respiration. Treatment with oligomycin allows the calculation of ATP production by the ATP synthase. **c** Representative glycolysis stress test showing extracellular acidification rate (ECAR) as a result of injection with 10 mM glucose, 1 μM oligomycin and 50 mM 2-deoxy-glucose (2DG). Response to glucose stimulation and relative maximal respiration are shown. All OCR and ECAR measurements were performed in sextuplicate and normalized to μg protein. Graphs show the mean are inclusive of at least three independent experiments. **d** Colony formation assay of E-Wnt, M-Wnt, metM-Wnt^lung^ cells grown in media containing 5 mM or 10 mM glucose or 10 mM galactose. Colonies were allowed to grow for 14 days before being fixed and stained. **e** Relative basal OCR and representative mitochondrial stress test of M6 and M6C cell lines. **f** Representative glycolysis stress test and relative glycolysis (ECAR) of M6 and M6C lines as compared with M6 cells. Graph shows mean ± SEM and is inclusive of three independent experiments. *p < 0.05, ***p < 0.001, Student’s *t*-test

As gene expression analyses of metM-Wnt^lung^ vs. M-Wnt cells showed changes in metabolic pathways, many converging on mitochondrial respiration (Supplemental Fig. 4), we conducted metabolic flux analysis using a Seahorse^®^ metabolic analyzer. Basal oxidative phosphorylation, as measured by oxygen consumption rate (OCR), was increased in metM-Wnt^lung^ cells compared with M-Wnt cells (Fig. 5a). Notably, E-Wnt cells displayed increased mitochondrial oxidative phosphorylation compared with M-Wnt cells. In contrast, metM-Wnt^lung^ cells displayed increased oxidative phosphorylation compared to M-Wnt cells, suggesting that M-Wnt cells have reduced mitochondrial respiration after EMT and that the respiration is regained as M-Wnt cells become metastatic. In a mitochondrial stress test, M-Wnt cells had significantly reduced mitochondrial respiration and respiratory capacity compared with E-Wnt and metM-Wnt^lung^ cells. No significant change in adenosine triphosphate (ATP) production was detected in any of the cell lines (Fig. 5b). M-Wnt cells exhibited increased glycolysis compared with E-Wnt cells (Fig. 5c), while metM-Wnt^lung^ cells displayed glycolytic activity not significantly different from E-Wnt or M-Wnt cells. M-Wnt cells displayed less metabolic adaptability, with reduced maximum glycolysis following ATP synthase inhibition by oligomycin.

Furthermore, M-Wnt, metM-Wnt^lung^ cells displayed significantly reduced growth in media containing low (5 mM) compared with high (25 mM) glucose, while E-Wnt colony growth was unchanged (Fig. 5d). When glucose was replaced with galactose, which does not support anaerobic respiration,^24^ metM-Wnt^lung^ cells formed as many colonies as in media with high glucose content. In contrast, M-Wnt cells formed fewer colonies using galactose as fuel source. Similar results were found in the metastatic cell line derived from liver metastases, metM-Wnt^liver^ (Supplemental Fig. 5). Taken together, these findings suggest that metM-Wnt^lung^ cells, relative to M-Wnt cells, have increased metabolic plasticity and decreased dependency on glycolysis, and thus can adapt to and compensate for changing fuel sources. Similar metabolic profiles were found in a second TNBC cellular progression series M6 cells, isolated from a spontaneous basal-like mammary adenocarcinoma from a C3TAg transgenic mouse, and M6C cells, derived from lung metastases following subcutaneous injection of the M6 line into a nude mouse.^25^ Metastatic M6C cells displayed increased OCR and extracellular acidification rate (ECAR) compared to nonmetastatic M6 cells (Fig. 5e and f). These results suggest that metabolic alterations are a feature of TNBC metastasis, and highlight metabolism as a potential target for the disease.

## Discussion

Aberrant Wnt signaling is a hallmark of TNBC.^26^ We previously established low-tumorigenic E-Wnt and high-tumorigenic M-Wnt cell lines that induce minimally invasive epithelial-like mammary tumors or highly invasive mesenchymal tumors, respectively.^16^ However, both E-Wnt and M-Wnt cells have low metastatic potential. Here, we established a metastatic tumor cell line (metM-Wnt^lung^) that shares the same genetic background as E-Wnt and M-Wnt cells but has high metastatic potential. metM-Wnt^lung^ cells retain the tumorigencity of their parental M-Wnt cells as well as many mesenchymal characteristics, including enhanced invasion and expression of Twist, TGF-β, and Slug. In addition, metM-Wnt cells contain an enriched CD44^high^/CD24^low^ population. While these markers have been used by some investigators to characterize a population of tumor cells with stem cell-like features, such markers are controversial, and it is not fully known if TICs arise through an EMT program, or are descendants of bipotent adult mammary cell progenitors. Indeed, the paucity of these bipotent progenitors in adult mice makes it difficult to trace TIC origins.^27^

metM-Wnt^lung^ cells also gain some epithelial characteristics, such as enhanced adhesion and tight colony formation, increased expression of E-cadherin and decreased expression of N-cadherin, Vimentin, Fibronectin, Oct4, and β-Catenin. These results are consistent with a progression model of metastasis involving EMT and MET,^6^ with cells underoing MET while still retaining mesenchymal characteristics.

Using our Wnt–driven progression series, now including metM-Wnt^lung^ cells, we identified a number of key genes and pathways altered between metastatic and nonmetastatic cells, including metabolic and inflammatory signals. These pathways were concordant between metM-Wnt^lung^ cells and metM-Wnt^liver^ cells, a second, independently derived metastatic TNBC cell line from transplanted M-Wnt cells. Using Oncomine meta-analysis, we identified 25 genes co-expressed or repressed in metM-Wnt cells and human metastatic breast cancers, including some in metabolic and inflammatory pathways, despite limited sample sizes and varied metastatic sites in human datasets. While efforts have been made to generate specific gene signatures for predicting metastatic disease, development of these gene signatures as targets for prevention or treatment is still lacking, and a deeper understanding of pathways altered during breast cancer metastasis is crucial.

We show that metabolism is altered during EMT and MET, with epithelial-like cells (E-Wnt) being highly oxidative, mesenchymal cells (M-Wnt) displaying reduced mitochondrial respiration and increased dependency on glycolysis, and metastatic cells (metM-Wnt^lung^ and metM-Wntl^iver^, M6C) displaying heightened mitochondrial and glycolytic energetics. No significant change in ATP production was detected across the cell lines, suggesting no defect in ATP synthase activity under normal conditions in M-Wnt cells. Recent studies have shown that cancer cells can derail metabolic pathways, simultaneously utilizing glycolysis and mitochondrial respiration through recycling nutrients such as glutamine and fatty acids to provide substrates.^28, 29^ In addition, we find several metabolic genes altered in M-Wnt cells compared with metM-Wnt^lung^ and metM-Wntl^iver^ cells, including genes associated with glycolysis, oxidative phosphorylation, adipogenesis, and MTORC1 signaling, as well as upstream signaling of metabolic pathways and solute carrier proteins. Of note, several genes associated with fat metabolism, including leptin, phytanoyl-CoA- hydroxylase (PHYH) and the fatty acid transporter FATP1. These results are consistent with recent work by Pascual et al.,^30^ showing that the fatty acid receptor CD36 is highly expressed in metastasis-initiating cells of oral carcinoma and that their metastatic potential is dependent on dietary fat to promote metastasis. Many of the altered genes and pathways in metM-Wnt cells converge on mitochondrial respiration, consistent with the metabolic flux analysis. Enhanced mitochondrial respiration during progression to metastasis is likely to provide ample ATP to fuel migration to, and colonization of, distant sites. Furthermore, nutrients at the site of metastasis are typically not limiting, unlike in the primary tumor. Thus, with increased availability of nutrients and oxygen at the metastatic site, the resumption of mitochondrial respiration is likely an efficient means of meeting the increased energy needs associated with metastasis.

While obesity is an established risk factor for TNBC development, the relationship between obesity and risk of metastasis is unclear, with the majority of research in the field being correlative.^12, 13^ We show directly that obese mice have enhanced metastatic TNBC tumor development compared with non-obese mice. These results are consistent with results from Kim et al.,^31^ in which a high-fat diet in non-ovariectomized mice resulted in enhanced growth and metastasis of the 4T1 mammary tumor cell line in the obesity-resistant FVB mouse model. Furthermore, we find that incubation of M-Wnt and metM-Wnt^lung^ cells with serum derived from obese mice, relative to serum from non-obese mice, caused significant change in expression of a number of genes associated with metabolism, including fatty acid metabolism, olfactory transduction, mTOR signaling and autophagy. These studies suggest that obesity-associated increases in TNBC metastasis may be due to metabolic changes. The crosstalk between intracellular metabolic changes within cancer cells and the systemic metabolic alterations in obese women may provide novel insights into how obesity contributes to breast cancer development and/or metastasis, and highlight potential treatment opportunities.

To our knowledge this is the first study utilizing a progression series derived from a single, nonmetastatic tumor origin to show metabolic alterations during each stage of metastatic development. Similar results have been seen in epithelial and mesenchymal human luminal breast cancer cell lines^32^ and murine TNBC cell lines.^33^ Metabolic reprogramming and plasticity has been described previously for lymph node-residing breast cancer cells,^34^ as well as brain,^35^ bone, lung, and liver metastases.^36^ Our findings of heightened mitochondrial respiration in Wnt and C3TAg metastases are consistent with findings by Dupuy et al., 2015.^36^ However, while this study revealed common metabolic alterations between metM-Wnt^lung^ and metM-Wnt^liver^ cells, Dupuy et al. found that high glycolysis and low oxidative phosphorylation was a predictor of liver metastasis and high oxidative phosphorylation was linked with lung and bone metastasis of 4T1 cells. This disparity may be due to genetic and other differences in the models, with the 4T1 tumor being an already highly metastatic tumor derived from a spontaneous tumor in a BALB/C mouse, while in this study we generated metastatic cell lines from a nonmetastatic parental M-Wnt cell line. Furthermore, the defined oncogenesis of the MMTV-Wnt1 C57BL/6 model^37^ may result in different metabolic signaling than that of the spontaneous tumor in BALB/C model.^38^

The relationship between energy metabolism and breast cancer metastasis has not been well studied. Increased mitochondrial activity in metastatic breast cancer cells seems to coincide with surrounding stromal cells becoming glycolytic, providing intermediates to the cancer cell in a process termed ‘reverse Warburg effect’.^34, 39^ These findings may also be cancer-specific, as reversion to a more oxidative state has been described for metastatic melanoma tumors,^39, 40^ while the highly metastatic osteosarcoma cell line DLM8-luc-M1 appears to be highly glycolytic.^41^ Based on findings presented here, we postulate that oxidative and nutrient stress occurring within a primary TNBC tumor fosters EMT progression and the shift from mitochondrial bioenergetics toward glycolytic energy production. Our mesenchymal, nonmetastatic M-Wnt cells have reduced metabolic plasticity and increased dependence on glucose for fuel. While glycolysis remains high following MET in metastatic TNBC, a partial reversion to an oxidative state in these cells facilitates more adaptability to changes in substrate availability.

In summary, we generated and characterized a metastatic murine TNBC cell line, extending our progression series of TNBC cell lines with shared genetics but differential tumorigenicity and metastatic potential. We identified transcriptomic and metabolic profiles underlying metastatic potential and examined the impact of obesity on metastasis. Our findings show that (1) metastatic cells display altered transcriptomic profiles compared to nonmetastatic cells, including several metabolic genes; (2) metastatic potential is associated with altered bioenergetics, including heightened glycolytic capacity and increased mitochondrial respiration; and (3) obesity increases metastatic potential, causing significant transcriptomic changes, particularly in metabolic pathways. To our knowledge, this is the first study to demonstrate altered metabolic plasticity during progression from nonmetastatic to metastatic TNBC lesion in a model derived from a common genetic background. Our findings suggest that targeting metabolic perturbations associated with metastatic potential is a novel strategy for reducing the burden of metastatic TNBC, particularly in obese women.

## Methods

### Animal care

All animal studies and procedures were approved and monitored by the Institutional Animal Care and Use Committee at University of North Carolina at Chapel Hill.

### Generation of metM-Wnt cell line

M-Wnt cells were transduced with a GFP-Luciferase dual labeling lentiviral construct (Systems Bioscience), and 5 × 10^5^ cells in 50 μl were injected into the tail vein or the fourth mammary fat pad of a SCID mouse. The resulting mammary fat pad tumor was excised and a tumor brei cell suspension was prepared as previously described,^16^ which was then injected into the 4th mammary fat pad of SCID mice (*n* = 3). For the survival surgery, mice were anesthetized by continuous flow of isoflurane, primary tumors were excised and incisions closed by wound clips. Mice were imaged biweekly by intraperitoneal injection of 200 μl D-Luciferin followed by isoflurane anesthesia and bioluminescence imaging using an IVIS Lumina II. Mice were sacrificed by CO_2_ inhalation followed by cervical dislocation and metastatic tumor explants were dissociated and propagated in vitro in Roswell Park Memorial Institute medium (RPMI)-1640 10% FBS, containing 25 mM glucose, 2 mM L-Glutamine and 100 U/ml penicillin/streptomycin and without added sodium pyruvate. GFP-positive metastatic cells were recovered by flow cytometry using a FACSAria III Cell Sorter (BD Biosciences). Prior to implantation into mice, cells were trypsinized and resuspended in serum-free RPMI-1640.

### In vitro characterization of metM-Wnt cell line

#### Colony formation

500 cells were seeded in triplicate into each well of a 6-well plate or 1000 cells suspended in a matrix of 0.4% agarose/culture media on a base of 0.8% agarose. Colonies were allowed to grow for up to 14 days before being fixed with methanol and stained with 0.5% crystal violet in 50% methanol. Colonies were imaged with a digital camera and counted.

#### Migration, invasion and adhesion assays

For invasion assays, 2.5 × 10^4^ cells were seeded in triplicate in serum-free RPMI-1640 media into Matrigel-coated invasion chambers (BD Biosciences, San Jose, CA) and allowed to invade (10% FBS as chemoattractant) for 18 h. Invasive cells were stained with 1% crystal violet in 50% methanol and counted using a light microscope. For adhesion assays, 96-well plates were coated with collagen I (0.01%) overnight at 4 °C and washed with PBS before being blocked with 2.5% bovine serum albumin for 2 h at 37 °C. Cells were suspended in serum-free RPMI at a density of 2.0 × 10^5^ cells/ml, seeded onto collagen-coated plates and allowed to attach for 30 min at 37 °C. Wells were washed once in PBS before the cells were fixed in methanol at −20 °C for 5 min. Cells were stained with 0.1% crystal violet and absorbance measured at 595 nm.

#### Flow cytometry

CD44^high^/CD24^low^ cell populations were analyzed by staining with CD44 and CD24 specific antibodies conjugated with BV421 and PE, respectively (BD Biosciences, San Jose, CA). Fluorescence intensity was measured using an LSR Fortessa (BD Biosciences).

### In vivo transplant studies

#### Tumorigenicity and metastatic potential of metM-Wnt^lung^ cells

Female albino C57BL/6 mice (*n* = 72) were randomized and transplanted with increasing concentrations of metM-Wnt cells into the fourth mammary fat pad (1 × 10^3^, 2.5 × 10^3^, 5 × 10^3^, 10 × 10^3^ 15 × 10^3^, 20 × 10^3^ cells in 50 μl per mouse; *n* = 12/group, G*Power analysis). Tumors were palpated twice weekly and mice were euthanized by CO_2_ inhalation followed by cervical dislocation when any tumor reached 1.5 cm in any one direction.

#### Diet study

Forty female ovariectomized albino C57BL/6 mice were singly housed and randomized to be fed either a DIO diet (60% kcal from fat; Research Diets # D12492) or a low fat, isonutrient matched control (10% kcal from fat; Research Diets # D12450J) ad libitum for 13 weeks before being harvested for body composition and fasting serum hormone analysis (4 h fasting, *n* = 5/group). Body fat and lean mass was measured using a Lunar Piximus X-Ray imager (GE Medical Systems, Ontario, CA). Serum hormones leptin, resistin and insulin were measured using a mouse diabetes multiplex assay on a MAGPIX multiplex reader (BioRad, Hercules, CA). The remaining mice received a tail vein injection of metM-Wnt^lung^ cells (2.5 × 10^3^/mouse in 200 μl; *n* = 15/group, G*Power analysis) and were maintained on their diet regimens. Mice were monitored carefully and were harvested upon signs of illness. Two mice from the DIO group were excluded upon being found dead in their cage. Incidence of macrometastases and micrometastases was determined by histological analysis and was validated by the Animal Histopathology Core at University of North Carolina at Chapel Hill.

### Tumor tissue staining

Excised tumor and lung tissue was fixed in 10% neutral-buffered formalin for 72 h, transferred to 70% ethanol and embedded in paraffin. Tissue blocks were cut into 4-μm thick sections for hematoxylin and eosin (H&E) or immunohistochemistry (IHC). Antigens were retrieved using a Tris-based buffer (pH 8.5) for 72 min at 100 °C, blocked with peroxidase for 8 min. Primary antibody (Ki67 anti-rabbit mAb (#D3B5; #12202, Cell Signaling, Danvers, MA) was applied (1:400) for 1 h, followed by secondary antibody (Omnimap, 760–4311) for 32 min. Slides were treated with DAB, Hematoxylin II for 12 min, and Bluing Reagent for 4 min. Staining was performed using Ventana’s Discovery Ultra Automated IHC system. Lung tissues were sectioned at 5 different planes 100 μm apart and stained with H&E and Ki67 to examine the extent of micrometastases throughout the lung.

### Gene expression microarray analysis

RNA was purified from cells from three extractions using RNeasy mini Kit (Qiagen, Hilden, Germany). cRNA libraries were generated using a Low Input Quick Amp labeling kit (Agilent Technologies, Santa Clara, CA) with NIH3T3 cells as reference RNA. Hybridizations were performed by the Lineberger Comprehensive Cancer Center Genomics Core, University of North Carolina as previously described^16^ using two-color 180 K Agilent microarrays (BARCODE25503) and scanned using an Agilent Technologies Scanner G2505C with Feature Extraction software (Santa Clara, CA). Arrays were then mean-adjusted and median-centered. For M-Wnt vs. metM-Wnt comprisons, transcripts with FC > 3 or FC < 0.33 and *q* < 0.001 by the SAM method were considered significant. Univariate statistics from all genes were used for pathway analysis along with the Hallmark gene sets on Molecular Signature Database (MSigDB) gene set enrichment analysis. Univariate statistics were summarized to a *z*-statistic for each gene set. Permutation was performed to assess significance of the gene set *z*-statistic. Altered genes in this dataset were compared with genes significantly altered in metastatic breast cancer samples relative to primary tumors (*p* < 0.05) from available human datasets.^18^ For in vitro obese vs. non-obese conditions, analysis was performed using a generalized linear model (glm) and a threshold of *p* < 0.001 was used to select candidate genes as none passed other testing criteria. To limit false discoveries, this list was further filtered by testing for enrichment using MSigDB. Microarray results have been deposited in the Gene Expression Omnibus (accession number GSE98703).

### Real-time PCR

RNA extractions, cDNA synthesis and EMT gene expression assays were performed as described previously.^16^ PCR and data collection were conducted on a ViiA7 (Applied Biosystems, Foster City, CA). Gene expression data were normalized to β-actin.

### Western immunoblot analysis

Cells were lysed in RIPA buffer. Forty micrograms of protein was loaded into a 4–15% stain-free gel (Biorad, Hercules, CA). Proteins were resolved and transferred to a PVDF membrane using a Transblot Turbo transfer unit (Biorad, Hercules, CA). Membranes were blocked with 5% bovine serum albumin for 1 h before being incubated overnight at 4 °C with one of the following primary antibodies: rabbit anti-E-Cadherin, rabbit anti-N-Cadherin (R & D Systems, Minneapolis MN), goat anti-Snail (Abcam, Cambridge, UK), rabbit anti-Beta-catenin (Abcam, Cambridge, UK) or mouse anti-β-Actin (Santa Cruz Biotechnology, Dallas, TX), followed by HRP-conjugated secondary antibodies raised against rabbit, mouse (Sigma Aldrich, St. Louis, MO) or goat IgG (Abcam). Proteins were visualized using a Chemi Doc MP system (Biorad, Berkeley, CA).

### Metabolic flux analysis

Cellular bioenergetics was determined using the Seahorse XF96 Analyzer (Agilent Seahorse Technologies, Santa Clara, CA). E-Wnt, M-Wnt, metM-Wnt^lung^, and metM-Wnt^liver^ cells were seeded at a concentration of 15 × 10^3^ cells per well in RPMI. M6 and M6C cells were seeded at a concentration of 10 × 10^3^ per well in Dulbecco's modified Eagle's Medium (DMEM). Prior to measurement, cells were incubated with basal RPMI (Gibco, Waltham, MA) or DMEM (Agilent Seahorse Technologies) supplemented with 2 mM glutamine, 10 mM glucose and 1 mM sodium pyruvate and incubated for 1 h at 37 °C in a CO_2_-free atmosphere. Basal OCR and ECAR were measured. OCR and ECAR responses were measured following administration of oligomycin (1 µM), carbonyl cyanide-4-(trifluormethoxy) phenylhydrazone (FCCP) (1 µM), and antimycin A (3 µM)/rotenone (3 µM) (XF Cell Mito Stress Test Kit, Agilent Seahorse Technologies). For glycolysis analysis, cells were incubated as above without glucose or sodium pyruvate and measurements were taken following administration of glucose (10 mM), oligomycin (1 µM) and 2-deoxyglucose (50 mM) (XF Glycolysis Stress Test, Agilent Seahorse Technologies). Results were normalized to μg protein using a BCA protein assay (Thermo Fisher, Waltham MA).

### Data analysis

Statistical analyses were performed using Graphpad Prism. Differences were assessed using the unpaired Student’s *t*-test or Gehan–Breslow–Wilcoxon test and results were considered significant if *p* < 0.05. R package samr was used for comparing M-Wnt vs. metM-Wnt^lung^ and metM-Wnt^liver^ cells.^42^ Genes with FC > 3 or FC < 0.33 and *q* < 0.001 were considered significant. For in vitro obese vs. non-obese comparisons, we used a generalized linear model and a threshold of *p* < 0.001.

## Electronic supplementary material

Supplemental Figure Legends

Supplemental File 4

Supplemental File 5

Supplemental File 1

Supplemental File 2

Supplemental File 3
