# Supplementary material for: Metabolic reprogramming underlies metastatic potential in an obesity-responsive murine model of metastatic triple negative breast cancer
Source: NPJ Breast Cancer. 2017 Jul 17;3:26. doi: 10.1038/s41523-017-0027-5 (PMC5514148; doi:10.1038/s41523-017-0027-5)
Supplement: Supplementary file 1 — Supplemental Figure Legends [file 41523_2017_27_MOESM1_ESM.docx]

**Supplemental File 1.** mRNA transcripts significantly altered in metastatic versus nonmetastatic M-Wnt cells. Changes were considered significantly different if *q<*0.001 and FC>3 or FC<0.33 (metM-Wnt^lung^/M-Wnt). Sheet 2 shows genes for our dataset that overlap with Oncomine datsets. Sheet 3 shows individual genes associated with pathways and overlap with human datasets.

**Supplemental File 2.** Heatmap showing mRNA transcripts altered in metM-Wnt^lung^ cells following incubation with obese or non-obese serum. Changes were considered significantly different if *p<*0.001.

**Supplemental File 3.** List of mRNA transcripts commonly altered in metM-Wnt^lung^ cells following incubation with obese or non-obese serum. Changes were considered significantly different if *p<*0.001.

**Supplemental File 4.** Schematic showing some of the most significant differentially expressed genes in metM-Wnt compared to M-Wnt cells, and their interactions with specific pathways found to be altered. Genes are shown in boxes with red denoting an increase in gene expression, and green denoting a decrease in gene expression. Pathways are shown in bold. Blue arrows show promotion, red lines denote inhibition. ALDH2 (aldehyde dehydrogenase 2); ANGPTL4 (angiopoeitin-like 4); ARTN (artemin); BCAA (branched chain amino acids); BCAT1 (branched chain aminotransferase 1); BDH2 (3-hydroxybutyrate dehydrogenase 2); CRABP2 (cellular retinoic binding protein 2); EDN1 (endothelian 1; ENO2 (enolase2); EPCR (endothelial protein C receptor); FA (fatty acid); FAS (fatty acid synthase); FATP1 (fatty acid transporter protein 1; SLC27A1); GSH (reduced gluthathione); GSTK1 (gluthathione-s-transferase kappa1); HIF1α (hypoxia inducible factor 1); HSD17B11 (hydroxysteroid (17 beta) dehydrogenase 11); ID2 (inhibitor of DNA binding 2); IL1R1 (interleukin 1 receptor type 1); ITGBL1 (integrin beta-like 1); LHPP (phospholysine phosphohistidine inorganic pyrophosphatase ); PGE_2_ (prostaglandin E_2_); PHYH (phytanol-CoA hydroxylase); PITPNC1 (phosphatidylinositol transfer protein cytoplasmic 1); PPAR (peroxisome proliferator-activated protein); Pi (inorganic phosphate); PTGES (prostaglandin E synthase); RRAD (Ras-related associated with diabetes); RRAGD (Ras-related GTP-binding protein D); RSG16 (regulator of G-protein signaling 16); SCL7A11 (solute carrier 7 member 11; cysteine-glutamate transporter); TGFBI (transforming growth factor beta induced).

**Supplemental File 5.** **Metabolic alterations in metM-Wnt^lung^ and metM-Wnt^liver^ cells compared to nonmetastatic TNBC cells.** (A) Mitochondrial and (B) glycolysis stress tests showing OCR and ECAR for E-Wnt, M-Wnt, metM-Wnt^lung^ and metM-Wnt^liver^ cells. (C) Colony formation assay showing growth of E-Wnt, M-Wnt, metM-Wnt^lung^ and metM-Wnt^liver^ cells in media containing 5 mM or 10 mM glucose or 10 mM galactose. **p*<0.05, ****p*<0.001, Student’s *t*-test.
