# Supplementary figures and images for: Metabolic reprogramming underlies metastatic potential in an obesity-responsive murine model of metastatic triple negative breast cancer
Source: NPJ Breast Cancer. 2017 Jul 17;3:26. doi: 10.1038/s41523-017-0027-5 (PMC5514148; doi:10.1038/s41523-017-0027-5)

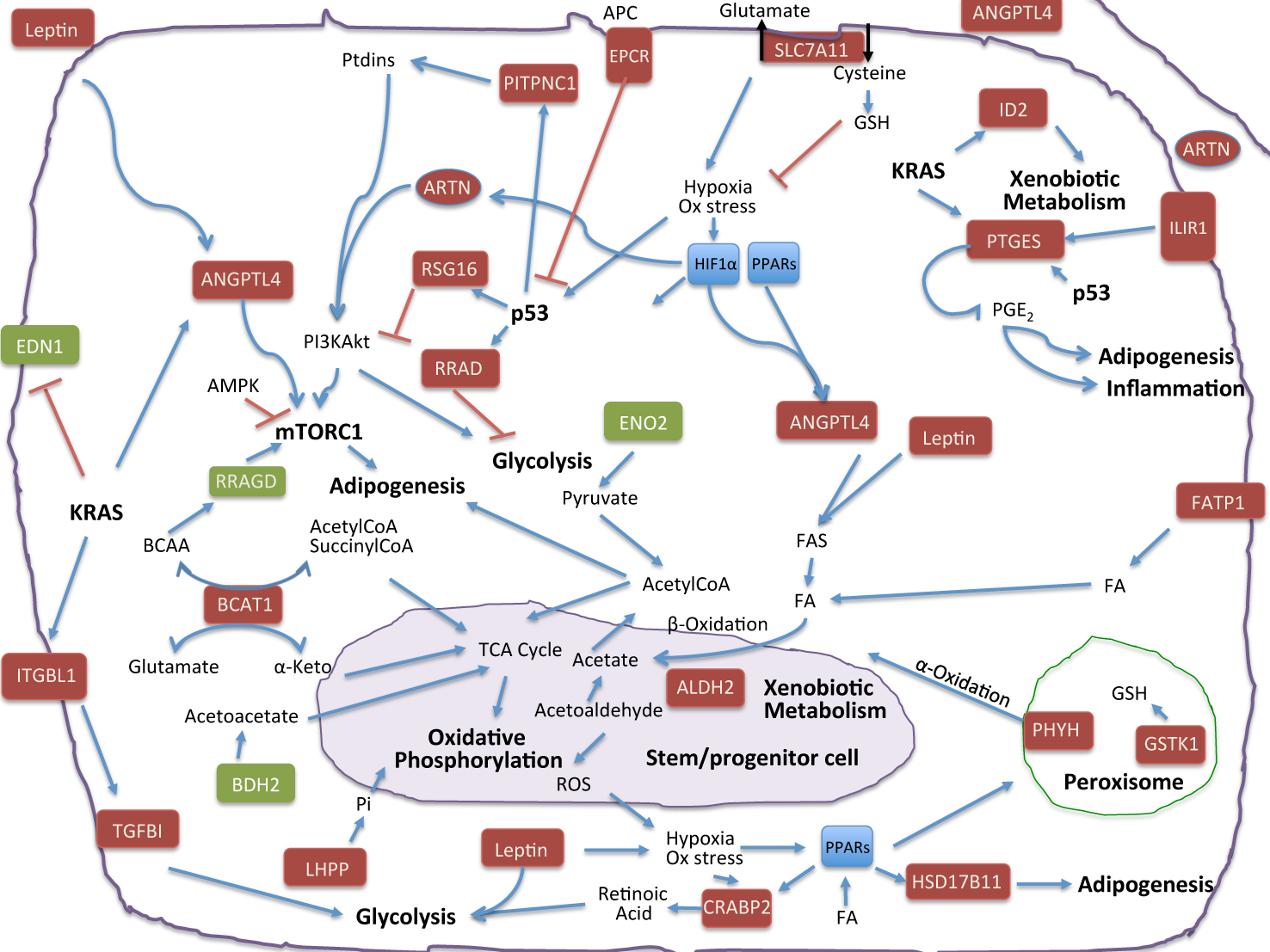

Supplement: Supplementary file 2 — Supplemental File 4 [file 41523_2017_27_MOESM2_ESM.tif]

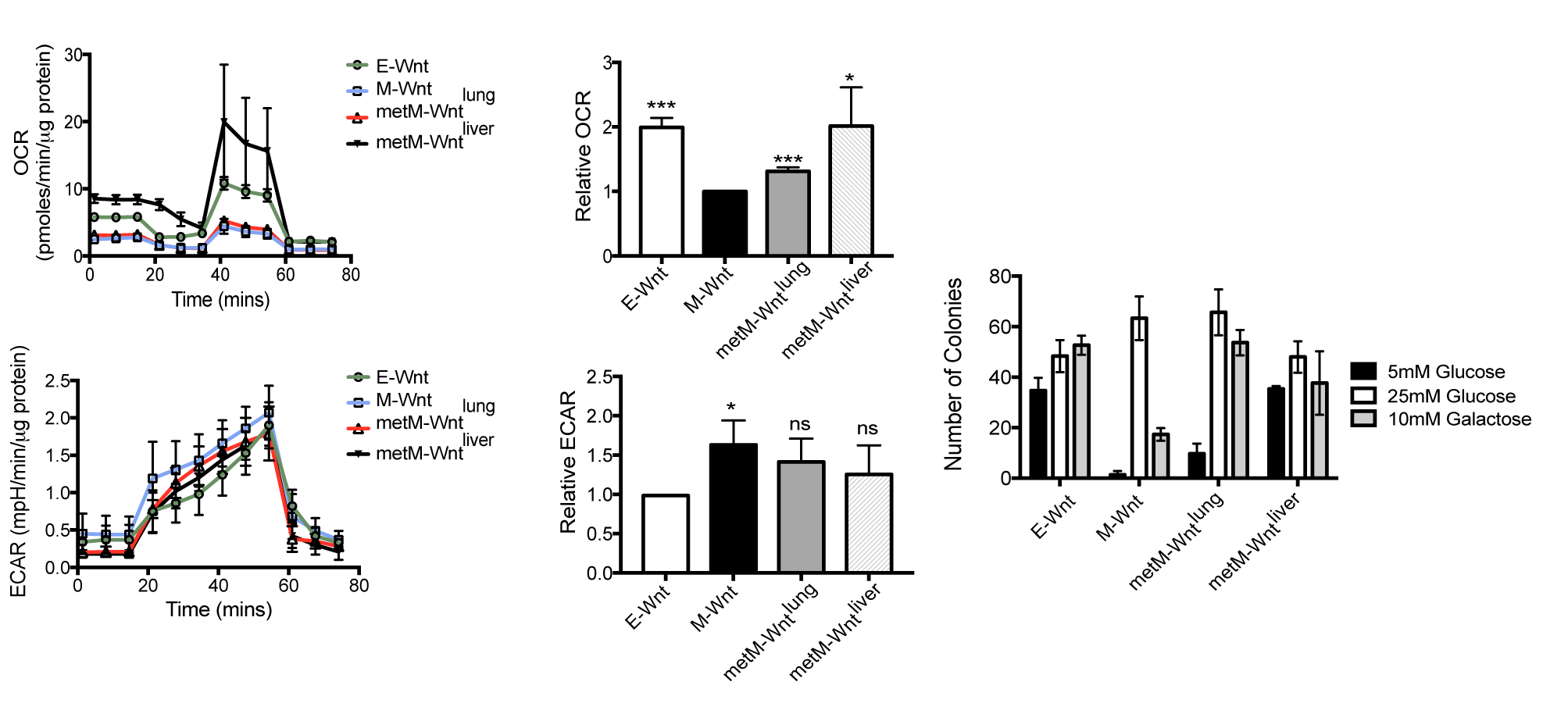

Supplement: Supplementary file 3 — Supplemental File 5 [file 41523_2017_27_MOESM3_ESM.tif]

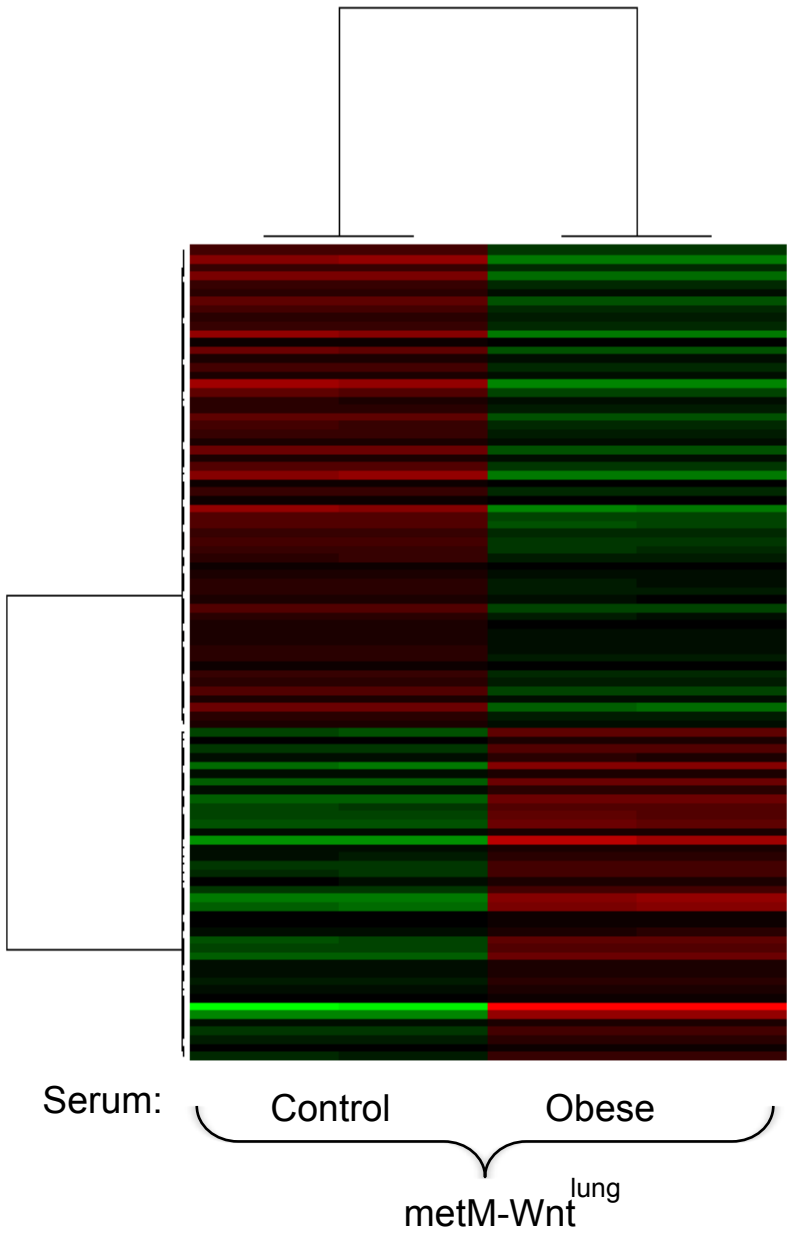

Supplement: Supplementary file 5 — Supplemental File 2 [file 41523_2017_27_MOESM5_ESM.pdf]
