# Supplementary material for: Metabolic reprogramming underlies metastatic potential in an obesity-responsive murine model of metastatic triple negative breast cancer
Source: NPJ Breast Cancer. 2017 Jul 17;3:26. doi: 10.1038/s41523-017-0027-5 (PMC5514148; doi:10.1038/s41523-017-0027-5)
Supplement: Supplementary file 4 — Supplemental File 1 [file 41523_2017_27_MOESM4_ESM.pdf]

| Gene Symb | Fold Chang | q-Value% | Hallmark Gene Pathway |
|---|---|---|---|
| GLIPR1 | 484.95024 | 0 | EMT |
| PIWIL4 | 198.84162 | 0 | |
| PIWIL2 | 196.75094 | 0 | |
| SERPINF1 | 106.06146 | 0 | |
| WNT10A | 82.899303 | 0 | |
| ESM1 | 76.621816 | 0 | |
| SPRY4 | 76.304797 | 0 | IL2/JAK/STAT5 |
| CRABP2 | 59.436026 | 0 | Peroxisome |
| IFITM1 | 58.489628 | 0 | Inflammatory response |
| INPP4B | 58.082726 | 0 | Androgen |
| ANKRD33B | 53.706698 | 0 | |
| CLEC1A | 52.114599 | 0 | |
| SIGIRR | 48.426534 | 0 | |
| ANGPTL4 | 44.23588 | 0 | Adipogenesis; Glycolysis; KRAS up |
| TSPAN11 | 43.912864 | 0 | |
| ITGBL1 | 42.465122 | 0 | KRAS up |
| CACNB2 | 41.487161 | 0 | |
| IL2RG | 39.783663 | 0 | KRAS up; Apical Surface; IL6/JAK/STAT3 |
| FAM73A | 37.981909 | 0 | |
| HRH1 | 37.361413 | 0 | Inflammatory response |
| ANKS1B | 36.794492 | 0 | |
| TBX20 | 34.588018 | 0 | |
| FAM159B | 33.013925 | 0 | |
| PCDH7 | 31.523523 | 0 | Inflammatory response |
| CD80 | 30.95929 | 0 | KRAS down; Allograft rejection; TNF |
| LPXN | 30.543722 | 0 | |
| NXF3 | 30.382359 | 0 | |
| PHLDA2 | 30.380546 | 0 | TNF |
| HAS1 | 30.362326 | 0 | |
| HSPA12A | 28.115459 | 0 | |
| TAF9B | 27.101062 | 0 | |
| CLEC4D | 27.019072 | 0 | |
| TSHZ3 | 26.555305 | 0 | |
| ENPP1 | 26.310373 | 0 | IL2/JAK/STAT5 |
| FAM132B | 26.114937 | 0 | |
| P2RY12 | 25.266721 | 0 | IFN Gamma |
| CCDC19 | 25.151033 | 0 | |
| SAMD12 | 25.115878 | 0 | |
| PTGES | 24.658923 | 0 | Xenobiotic metabolism |
| DDIT4L | 24.248061 | 0 | |
| EDN2 | 23.500443 | 0 | KRAS down |

| Gene Symbo | Fold Chang | q-Value% | Hallmark Gene Pathway |
|---|---|---|---|
| FGF21 | 23.261294 | 0 | |
| SLC9A2 | 23.04862 | 0 | |
| CNTNAP4 | 22.401792 | 0 | |
| ARL11 | 21.853644 | 0 | |
| PDE6H | 21.851167 | 0 | |
| HGF | 20.525663 | 0 | Apoptosis |
| SLCO4A1 | 20.437985 | 0 | |
| HHIP | 18.854451 | 0 | |
| AFF2 | 18.646953 | 0 | |
| HMX2 | 18.631311 | 0 | |
| PTPN22 | 18.358771 | 0 | |
| PTGS1 | 18.304078 | 0 | |
| NRARP | 18.191814 | 0 | |
| IGFBP6 | 17.671221 | 0 | Apoptosis |
| PTHLH | 17.580523 | 0 | EMT |
| TMEM16A | 17.369343 | 0 | |
| LONRF3 | 17.200781 | 0 | |
| RGS16 | 16.935903 | 0 | p53; KRAS up; Inflammation; IL2/JAK/STAT5 |
| CXCR6 | 16.156062 | 0 | Inflammatory response |
| ABCG1 | 15.978151 | 0 | |
| EPB41L4A | 15.934769 | 0 | |
| ARHGDIB | 15.877426 | 0 | |
| SLAMF8 | 15.561823 | 0 | |
| ECSCR | 15.237282 | 0 | |
| SMPD5 | 14.434054 | 0 | |
| CER1 | 14.110396 | 0 | |
| TLK1 | 14.062576 | 0 | |
| SOBP | 13.985308 | 0 | |
| EXOC3L4 | 13.88281 | 0 | |
| SLC38A1 | 13.781811 | 0 | |
| SLC16A4 | 13.71867 | 0 | |
| KCNF1 | 13.579375 | 0 | |
| CENTD3 | 13.362589 | 0 | |
| TMPRSS5 | 13.246471 | 0 | |
| HPDL | 12.997633 | 0 | |
| EPSTI1 | 12.985716 | 0 | IFN Gamma |
| FRMD5 | 12.90898 | 0 | |
| SH2D1B2 | 12.758073 | 0 | |
| CSGALNAC | 12.752363 | 0 | |
| RASL10A | 12.558329 | 0 | |
| TMEM119 | 12.4826 | 0 | |

| Gene Symb | Fold Chang | q-Value% | Hallmark Gene Pathway |
|---|---|---|---|
| LTBP2 | 12.419609 | 0 | TGFBeta |
| TGFB1I1 | 12.371164 | 0 | |
| CYP2D22 | 12.296832 | 0 | |
| CHST7 | 12.170462 | 0 | |
| HSPA12B | 12.165794 | 0 | |
| RANGRF | 12.075319 | 0 | |
| BNC1 | 12.007666 | 0 | |
| PLAC9 | 11.977009 | 0 | |
| SCRN1 | 11.951777 | 0 | |
| C1QDC2 | 11.93813 | 0 | |
| AIG1 | 11.8939 | 0 | |
| LRRTM2 | 11.819562 | 0 | |
| ENOX1 | 11.734122 | 0 | |
| BCAR3 | 11.676804 | 0 | TGFBeta |
| IL15 | 11.626879 | 0 | EMT; Inflammatory response; IFN Gamma |
| CGREF1 | 11.418549 | 0 | |
| CAR12 | 11.296919 | 0 | |
| USP43 | 11.24882 | 0 | |
| CRIP2 | 11.19056 | 0 | Coagulation |
| ANGPT2 | 11.12624 | 0 | Adipogenesis |
| CD38 | 10.867237 | 0 | Apoptosis; IL6/JAK/STAT3; IFN Gamma |
| HSD17B11 | 10.78296 | 0 | Peroxisome |
| B3GNT3 | 10.570511 | 0 | Glycolysis |
| SEMA4D | 10.433402 | 0 | Inflammatory response |
| EMB | 10.393792 | 0 | |
| COL18A1 | 10.317342 | 0 | |
| GPR149 | 10.178222 | 0 | |
| GATM | 10.026781 | 0 | |
| SAMD5 | 9.9780603 | 0 | |
| PODNL1 | 9.8694015 | 0 | |
| DUSP2 | 9.8588634 | 0 | |
| CLMP | 9.7901382 | 0 | |
| CAMK1D | 9.7429658 | 0 | KRAS down |
| EOMES | 9.5714867 | 0 | IL2/JAK/STAT5 |
| TRERF1 | 9.5603402 | 0 | |
| FAM69C | 9.5421696 | 0 | |
| ZIC5 | 9.3305118 | 0 | |
| S100A7A | 9.1638684 | 0 | |
| ITGA10 | 9.1095524 | 0 | |
| PADI4 | 9.1054114 | 0 | |
| TRAF1 | 8.9838245 | 0 | KRAS up; Apical junction; IL2/JAK/STAT5 |

| Gene Symb[illegible] | Fold Chang[illegible] | q-Value% | Hallmark Gene Pathway |
|---|---|---|---|
| GPR123 | 8.9247982 | 0 | |
| ARTN | 8.8581517 | 0 | Glycolysis |
| CLDN1 | 8.7987569 | 0 | |
| AS3MT | 8.7133169 | 0 | |
| NID1 | 8.7105608 | 0 | |
| LHPP | 8.6748703 | 0 | Glycolysis |
| TRPV3 | 8.6614518 | 0 | |
| GLT8D2 | 8.5081958 | 0 | |
| ID2 | 8.4877295 | 0 | KRAS up; Xenobiotic metabolism; TGFBeta; [illegible] |
| MYCN | 8.3907305 | 0 | KRAS up |
| CPNE8 | 8.3893091 | 0 | |
| SGSM1 | 8.3725728 | 0 | |
| PCDH10 | 8.3673871 | 0 | |
| COL6A3 | 8.3661018 | 0 | EMT |
| SH3GL3 | 8.1851374 | 0 | |
| SPNS2 | 8.1452086 | 0 | |
| MYBBP1A | 8.1304582 | 0 | |
| QPCT | 8.110025 | 0 | |
| ODZ3 | 8.0812586 | 0 | |
| PEAR1 | 8.0119403 | 0 | |
| DEPDC6 | 7.9755433 | 0 | |
| HMHA1 | 7.8819595 | 0 | |
| MMP2 | 7.856226 | 0 | EMT; Apical Junction; Apoptosis; Coagulation |
| IRF8 | 7.8253882 | 0 | KRAS up; Xenobiotic metabolism; IFN Gamm[illegible] |
| PDGFRA | 7.8249688 | 0 | |
| IL1R2 | 7.6584568 | 0 | Xenobiotic metabolism; IL6/JAK/STAT3; IL2/J[illegible] |
| CXCL14 | 7.6550291 | 0 | |
| EGR3 | 7.591274 | 0 | Apoptosis; TNF |
| ARHGAP27 | 7.5118829 | 0 | |
| OMD | 7.5105208 | 0 | Adipogenesis |
| IGSF10 | 7.4494835 | 0 | |
| NGEF | 7.4489244 | 0 | |
| ABCB1A | 7.3727345 | 0 | |
| MGAT3 | 7.3698033 | 0 | |
| DOCK10 | 7.3261336 | 0 | |
| NQO1 | 7.3258634 | 0 | Xenobiotic metabolism |
| PRODH | 7.2937562 | 0 | KRAS down |
| CARD10 | 7.2449914 | 0 | |
| PHYH | 7.2366976 | 0 | Adipogenesis; Oxidative phosphorylation |
| RRAD | 7.2082075 | 0 | p53; UV |
| TFPI | 7.1324188 | 0 | KRAS up |

| Gene Symb | Fold Chang | q-Value% | Hallmark Gene Pathway |
|---|---|---|---|
| SEC16B | 7.124904 | 0 | |
| NT5E | 7.0827718 | 0 | Glycolysis; EMT; IL2/JAK/STAT5 |
| KLK6 | 7.0522804 | 0 | |
| CALCRL | 6.9931798 | 0 | Inflammatory response |
| PRICKLE1 | 6.9744626 | 0 | |
| HAVCR2 | 6.869423 | 0 | |
| SCIN | 6.8693796 | 0 | |
| RBPMS | 6.8590156 | 0 | |
| ODF3L1 | 6.8564668 | 0 | |
| PROCR | 6.8179742 | 0 | p53 |
| LEPREL1 | 6.7924002 | 0 | |
| ANXA8 | 6.6845936 | 0 | |
| LGI2 | 6.660762 | 0 | |
| SOD3 | 6.6536218 | 0 | |
| TNFSF11 | 6.6525317 | 0 | IL2/JAK/STAT5 |
| HIST1H1A | 6.5798491 | 0 | p53 |
| KALRN | 6.5640039 | 0 | |
| CHDH | 6.5052624 | 0 | |
| CAR5B | 6.4796933 | 0 | |
| FAM89A | 6.464202 | 0 | |
| ANKRD34A | 6.4594819 | 0 | |
| LTC4S | 6.4473224 | 0 | Adipogenesis |
| DNAJC22 | 6.4029423 | 0 | |
| CXCR7 | 6.3959401 | 0 | TNF |
| KCNK10 | 6.3000581 | 0 | |
| MYCL1 | 6.2979789 | 0 | |
| TRIM66 | 6.2875498 | 0 | |
| CXXC5 | 6.2433122 | 0 | |
| TMEM74 | 6.2366706 | 0 | |
| MTUS1 | 6.2320142 | 0 | |
| SCML4 | 6.1913985 | 0 | |
| LMO7 | 6.1881878 | 0 | |
| MYL9 | 6.1835411 | 0 | EMT; Apical Junction |
| DIXDC1 | 6.1377459 | 0 | |
| PPEF2 | 6.1136195 | 0 | |
| ARHGEF15 | 6.0895206 | 0 | |
| MAB21L3 | 6.0497978 | 0 | |
| APOB48R | 6.028717 | 0 | |
| MRGPRF | 6.0160407 | 0 | |
| PLEC1 | 5.9706543 | 0 | |
| PHYHIPL | 5.9350169 | 0 | |

| Gene Symbo | Fold Chang | q-Value% | Hallmark Gene Pathway |
|---|---|---|---|
| RASSF6 | 5.8676409 | 0 | |
| ARHGAP6 | 5.8584381 | 0 | |
| SEMA6D | 5.8424914 | 0 | |
| PVT1 | 5.8344584 | 0 | p53 |
| FAM19A1 | 5.8232353 | 0 | |
| CD244 | 5.8067335 | 0 | |
| JAG1 | 5.7977066 | 0 | Notch; Wnt/BetaCatenin; Angiogenesis; TNF |
| MMD | 5.7952878 | 0 | KRAS up |
| JPH2 | 5.7172717 | 0 | |
| GSTK1 | 5.701567 | 0 | Peroxisome |
| FOXS1 | 5.6982924 | 0 | |
| GSDMD | 5.6510923 | 0 | |
| USP2 | 5.6501077 | 0 | |
| ABHD15 | 5.6312953 | 0 | |
| FAM20B | 5.6247387 | 0 | |
| KCTD10 | 5.6142819 | 0 | |
| PLEK2 | 5.6074938 | 0 | KRAS up |
| SLC27A1 | 5.6054709 | 0 | Adipogenesis |
| EPAS1 | 5.5789778 | 0 | |
| SPINK2 | 5.5601732 | 0 | |
| EID2 | 5.5415385 | 0 | |
| TTC12 | 5.5198707 | 0 | |
| POU2AF1 | 5.4865349 | 0 | |
| EMILIN1 | 5.4757773 | 0 | |
| ANK | 5.4295502 | 0 | |
| ACPP | 5.4101602 | 0 | |
| DNMT3L | 5.4037591 | 0 | |
| CHRNA1 | 5.3928755 | 0 | |
| CCDC74A | 5.3918501 | 0 | |
| CYB561 | 5.3756312 | 0 | |
| PLAT | 5.3684712 | 0 | KRAS up; Apoptosis; Coagulation |
| GDF15 | 5.3672616 | 0 | |
| CACNA1C | 5.3443565 | 0 | |
| MAF | 5.3220085 | 0 | Androgen |
| SPN | 5.3108012 | 0 | |
| POPDC3 | 5.2258432 | 0 | |
| PPBP | 5.224811 | 0 | KRAS up |
| EVI2B | 5.2200096 | 0 | |
| COL3A1 | 5.2133645 | 0 | EMT |
| LAT2 | 5.195537 | 0 | KRAS up |
| SYT12 | 5.1877216 | 0 | |

| Gene Symb | Fold Chang | q-Value% | Hallmark Gene Pathway |
| --- | --- | --- | --- |
| GUCA1A | 5.1654766 | 0 | |
| SLC1A1 | 5.1593883 | 0 | |
| KCNAB2 | 5.151828 | 0 | |
| LRRC14 | 5.1412691 | 0 | |
| LPHN3 | 5.1155405 | 0 | |
| FLRT3 | 5.1139561 | 0 | |
| BAALC | 5.1134327 | 0 | |
| NOL3 | 5.0996757 | 0 | Glycolysis |
| NES | 5.0803327 | 0 | |
| TXK | 5.0696096 | 0 | |
| BOC | 5.0636744 | 0 | |
| FAM81A | 5.0579197 | 0 | |
| PRICKLE2 | 5.0508116 | 0 | |
| SYT13 | 5.0373323 | 0 | |
| C13orf15 | 5.0260572 | 0 | |
| SMG6 | 5.0219058 | 0 | |
| PLAU | 5.0157135 | 0 | KRAS up; Coagulation |
| CLN3 | 4.9885261 | 0 | |
| CD248 | 4.9447817 | 0 | |
| TBC1D2 | 4.9418631 | 0 | |
| ITGB3 | 4.937394 | 0 | EMT; Inflammation; Coagulation; IL6/JAK/ST |
| MFSD2 | 4.9370681 | 0 | |
| CKAP4 | 4.9294479 | 0 | IL2/JAK/STAT5 |
| NOSTRIN | 4.9210126 | 0 | |
| PLA2G7 | 4.9095649 | 0 | |
| DLX3 | 4.9029915 | 0 | |
| HSPB1 | 4.8638037 | 0 | Apoptosis; Apical Surface |
| IL1RL1 | 4.851247 | 0 | IL2/JAK/STAT5 |
| MURC | 4.8319848 | 0 | |
| ITGB7 | 4.821378 | 0 | IFN Gamma |
| IL1R1 | 4.8017588 | 0 | Inflammatory response; IL6/JAK/STAT3 |
| TNFRSF13C | 4.7943298 | 0 | |
| CHST2 | 4.7937173 | 0 | Glycolysis; KRAS down; Inflammatory respon |
| DCLK1 | 4.7742912 | 0 | |
| IPMK | 4.7675924 | 0 | |
| TRPV2 | 4.7043549 | 0 | |
| LYNX1 | 4.7005332 | 0 | |
| TMEFF2 | 4.695465 | 0 | |
| CBX7 | 4.6874827 | 0 | |
| NPY5R | 4.6812396 | 0 | |
| RNF165 | 4.678325 | 0 | |

| Gene Symbo | Fold Chang | q-Value% | Hallmark Gene Pathway |
|---|---|---|---|
| SLC35F2 | 4.6308028 | 0 | |
| PRKCB1 | 4.6038709 | 0 | |
| IL11 | 4.5792482 | 0 | Allograft rejection |
| BLNK | 4.5765655 | 0 | |
| DHH | 4.5460378 | 0 | |
| AHR | 4.5316834 | 0 | Inflammatory response; IL2/JAK/STAT5 |
| DNM1 | 4.5078646 | 0 | |
| EIF4E3 | 4.5056539 | 0 | IFN Gamma |
| NPPB | 4.5040408 | 0 | |
| AKNAD1 | 4.5031472 | 0 | |
| RGNEF | 4.4978906 | 0 | |
| SEMA3B | 4.4709664 | 0 | KRAS up |
| RAMP3 | 4.4654638 | 0 | |
| DCDC2C | 4.4621279 | 0 | |
| JAG2 | 4.4322984 | 0 | p53; Wnt/Beta Catenin; Angiogenesis |
| LEPR | 4.4016706 | 0 | IL6/JAK/STAT3 |
| PPP1R9A | 4.3995821 | 0 | |
| DLGAP1 | 4.3766435 | 0 | |
| RILPL1 | 4.3683765 | 0 | |
| S100B | 4.3674323 | 0 | |
| SPNS3 | 4.3662754 | 0 | |
| PKD2 | 4.3522398 | 0 | |
| DHRS9 | 4.3142492 | 0 | |
| HS3ST3A1 | 4.3056857 | 0 | |
| TTC39C | 4.2922803 | 0 | |
| SLC4A11 | 4.271787 | 0 | |
| PLXDC1 | 4.2620154 | 0 | |
| CABYR | 4.2598151 | 0 | |
| BTC | 4.2369621 | 0 | KRAS up |
| SUHW4 | 4.2358373 | 0 | |
| C1ORF54 | 4.2299576 | 0 | |
| FHOD3 | 4.2266509 | 0 | |
| PSCDBP | 4.2246168 | 0 | |
| P2RY14 | 4.2218419 | 0 | |
| NPAS4 | 4.1774318 | 0 | |
| SORBS1 | 4.1743746 | 0 | Adipogenesis |
| FYB | 4.1695464 | 0 | Apical Junction; Allograft rejection |
| ADAM8 | 4.1566519 | 0 | KRAS up |
| TNFAIP6 | 4.1490579 | 0 | Inflammatory response; IFN Gamma |
| EPHX2 | 4.1483117 | 0 | Adipogenesis; Peroxisome |
| TMEM141 | 4.1381008 | 0 | |

| Gene Symbo | Fold Chang | q-Value% | Hallmark Gene Pathway |
|---|---|---|---|
| VCAN | 4.1357331 | 0 | Glycolysis; EMT; Apical junction; Angiogenesis |
| ANKK1 | 4.1244426 | 0 | |
| ARHGAP30 | 4.1135016 | 0 | |
| LY6E | 4.0870874 | 0 | Inflammatory response; IFN Gamma |
| ALDH2 | 4.0750575 | 0 | Adipogenesis; Xenobiotic metabolism |
| SEC1 | 4.0675742 | 0 | |
| RPS6KA2 | 4.0633889 | 0 | |
| CORO2A | 4.0567146 | 0 | |
| SORCS2 | 4.056639 | 0 | |
| ST3GAL1 | 4.0381412 | 0 | |
| RAB13 | 4.0346592 | 0 | |
| CRLF1 | 4.0332498 | 0 | EMT |
| JHDM1D | 4.0270867 | 0 | |
| C1QTNF1 | 4.0195568 | 0 | |
| KRR1 | 4.0071438 | 0 | |
| SYT7 | 3.9984045 | 0 | |
| GRHL2 | 3.9946946 | 0 | |
| ACSBG1 | 3.9841205 | 0 | |
| HMCN2 | 3.9826082 | 0 | |
| APOBEC3 | 3.9450317 | 0 | |
| CCDC37 | 3.9446417 | 0 | |
| GCNT1 | 3.9323781 | 0 | Allograft rejection |
| SOAT2 | 3.9261711 | 0 | |
| IL18R1 | 3.9233104 | 0 | Inflammatory response; IL6/JAK/STAT3; IL2/J |
| FBLN1 | 3.9214191 | 0 | Xenobiotic metabolism; EMT |
| GPR124 | 3.9119498 | 0 | KRAS up |
| MRC1 | 3.9067183 | 0 | |
| CCDC81 | 3.9021063 | 0 | |
| TRAPPC9 | 3.9017481 | 0 | |
| MAMSTR | 3.8691639 | 0 | |
| SNCG | 3.8642882 | 0 | Adipogenesis |
| S100A16 | 3.857488 | 0 | |
| ELA1 | 3.8477817 | 0 | |
| GNB4 | 3.8388158 | 0 | |
| LHX9 | 3.8385599 | 0 | Glycolysis |
| PIK3R5 | 3.8000154 | 0 | Inflammation; IL6/JAK/STAT3 |
| THBS1 | 3.7963123 | 0 | EMT; TGFBeta; Coagulation |
| PITPNC1 | 3.7934465 | 0 | p53 |
| AMIGO1 | 3.7880283 | 0 | Apical Junction |
| DNAHC1 | 3.7794741 | 0 | |
| PRSS16 | 3.7750591 | 0 | |

| Gene Symbol | Fold Change | q-Value% | Hallmark Gene Pathway |
|---|---|---|---|
| SLC7A11 | 3.7645879 | 0 | p53; MTORC1 |
| ERGIC1 | 3.7572469 | 0 | |
| PTGER4 | 3.747491 | 0 | Inflammation; TNF |
| BAIAP2L1 | 3.7472385 | 0 | |
| HPSE | 3.7301907 | 0 | |
| PSD4 | 3.7257379 | 0 | |
| RBPMS2 | 3.7251887 | 0 | |
| HIST1H1C | 3.7103482 | 0 | |
| CBR3 | 3.7045875 | 0 | |
| KHDRBS3 | 3.6868851 | 0 | |
| ODZ4 | 3.6604608 | 0 | |
| OLFM1 | 3.656213 | 0 | UV |
| NKAPL | 3.6560696 | 0 | |
| ZC3H14 | 3.6493149 | 0 | |
| ATP2C2 | 3.6365148 | 0 | |
| EML5 | 3.6363951 | 0 | |
| FIGF | 3.6302025 | 0 | |
| ARMC2 | 3.6129085 | 0 | |
| CTNNAL1 | 3.6026907 | 0 | |
| GLIS1 | 3.6000163 | 0 | |
| ARHGAP28 | 3.5880089 | 0 | |
| TGFBI | 3.5756274 | 0 | Glycolysis; Apical junction; EMT |
| APOL6 | 3.5713738 | 0 | IFN Gamma |
| DMP1 | 3.5560984 | 0 | Apical Junction |
| MGST2 | 3.5498178 | 0 | |
| COL12A1 | 3.5461211 | 0 | EMT |
| KIF26B | 3.5452909 | 0 | |
| TMEM154 | 3.5340908 | 0 | |
| SOX11 | 3.520639 | 0 | |
| NPM2 | 3.507678 | 0 | |
| ANGPT1 | 3.4868229 | 0 | |
| STAC2 | 3.4751413 | 0 | |
| RTN4RL1 | 3.4739242 | 0 | Apical Surface |
| BFSP1 | 3.472117 | 0 | |
| ASPN | 3.4700679 | 0 | |
| F2R | 3.4385813 | 0 | p53; Apoptosis; Allograft rejection |
| CD109 | 3.4368057 | 0 | |
| PROKR1 | 3.4335968 | 0 | |
| DOCK8 | 3.424168 | 0 | |
| ESYT2 | 3.4209538 | 0 | |
| SGCD | 3.4100588 | 0 | EMT |

| Gene Symb | Fold Chang | q-Value% | Hallmark Gene Pathway |
|---|---|---|---|
| DAB2 | 3.4070339 | 0 | EMT |
| MYO10 | 3.4026196 | 0 | |
| PTPN13 | 3.3997253 | 0 | |
| RNASEN | 3.3989918 | 0 | |
| PHEX | 3.3982451 | 0 | |
| NFE2L3 | 3.3926741 | 0 | |
| Gene Symb | Fold Chang | q-Value% | Hallmark Gene Pathway |
| ENPP3 | 3.3806826 | 0 | |
| DUSP18 | 3.3774993 | 0 | TNF |
| FGF1 | 3.3770322 | 0 | |
| SCFD2 | 3.3734814 | 0 | |
| PPAP2A | 3.3680167 | 0 | Androgen; IL2/JAK/STAT5 |
| PTPRE | 3.3615941 | 0 | p53; Inflammatory response |
| SHROOM1 | 3.3531182 | 0 | |
| ASPH | 3.3438476 | 0 | |
| GARNL4 | 3.3383634 | 0 | |
| MBNL2 | 3.3383449 | 0 | |
| NAAA | 3.3380646 | 0 | |
| RALGPS2 | 3.3302867 | 0 | |
| GLP1R | 3.329114 | 0 | |
| OAF | 3.3257727 | 0 | |
| CTGF | 3.3248678 | 0 | EMT |
| NOTUM | 3.3150776 | 0 | |
| PTGFRN | 3.3076427 | 0 | |
| TDRD5 | 3.3058415 | 0 | |
| CENTD1 | 3.3056576 | 0 | |
| UBD | 3.2991969 | 0 | |
| MYH9 | 3.2969163 | 0 | Hedgehog; Apical Junction |
| SLC25A32 | 3.286509 | 0 | |
| TATDN1 | 3.2830556 | 0 | |
| FAIM3 | 3.2802245 | 0 | |
| SEPT5 | 3.2801161 | 0 | |
| PRSS46 | 3.2799536 | 0 | |
| TBKBP1 | 3.2798016 | 0 | |
| EFR3A | 3.2717377 | 0 | |
| GGT1 | 3.2705313 | 0 | |
| GP1BB | 3.2659406 | 0 | |
| CABP4 | 3.2583176 | 0 | |
| SBSN | 3.2554992 | 0 | |
| BCAT1 | 3.2515365 | 0 | MTORC1; Xenobiotic metabolism; Allograft re |
| RGL1 | 3.2472461 | 0 | |

| Gene Symb | Fold Chang | q-Value% | Hallmark Gene Pathway |
|---|---|---|---|
| CENTB1 | 3.2266189 | 0 | |
| RASGRP3 | 3.2204691 | 0 | |
| ITGA1 | 3.2201552 | 0 | Apical Junction |
| ARHGAP9 | 3.2182944 | 0 | |
| ADH7 | 3.2169198 | 0 | Xenobiotic metabolism |
| GNG2 | 3.2139411 | 0 | |
| NPNT | 3.2126944 | 0 | |
| TUBB2B | 3.1979862 | 0 | |
| REEP6 | 3.1978486 | 0 | Adipogenesis |
| ERN1 | 3.1957887 | 0 | Unfolded Protein Response |
| CCL27 | 3.1927148 | 0 | |
| PRND | 3.1926645 | 0 | |
| LHX1 | 3.1808321 | 0 | |
| BEND6 | 3.1769853 | 0 | |
| GNG8 | 3.1581705 | 0 | |
| RGS14 | 3.1487466 | 0 | |
| ACOT6 | 3.1444731 | 0 | |
| STAB1 | 3.1393806 | 0 | Inflammatory response response; Allograft rej |
| CD97 | 3.1351328 | 0 | |
| CSPG4 | 3.1248269 | 0 | |
| NRCAM | 3.1107819 | 0 | Hedgehog |
| ICOSL | 3.1006053 | 0 | |
| ENTHD1 | 3.0903376 | 0 | |
| COTL1 | 3.0836195 | 0 | |
| DPEP1 | 3.0825055 | 0 | |
| PAQR7 | 3.0820517 | 0 | |
| NKX6-1 | 3.068766 | 0 | Hedgehog |
| C11orf88 | 3.067419 | 0 | |
| HIC1 | 3.0570251 | 0 | |
| IFITM3 | 3.0508405 | 0 | Apoptosis; IFN Gamma |
| PDLIM1 | 3.0500106 | 0 | |
| SYNJ2 | 3.0476871 | 0 | |
| ELOVL3 | 3.0473302 | 0 | |
| POP1 | 3.0428283 | 0 | |
| GTSF1 | 3.0334528 | 0 | |
| ARHGAP8 | 3.0304069 | 0 | |
| HAS2 | 3.0300784 | 0 | Inflammatory response response |
| FGF11 | 3.0210139 | 0 | |
| ASS1 | 3.0105754 | 0 | |
| LRP12 | 3.0080596 | 0 | |
| F3 | 3.0020373 | 0 | Inflammatory response response; Coagulatio |

| Gene Symbo | Fold Change | q-Value% | Hallmark Gene Pathway |
|---|---|---|---|
| THEM2 | 0.3329043 | 0 | |
| SAA4 | 0.3315599 | 0 | |
| ENO2 | 0.3307737 | 0 | Glycolysis; EMT; UV; Apoptosis |
| GSC | 0.3289575 | 0 | |
| HSD17B1 | 0.3288278 | 0 | |
| TBC1D9 | 0.3268964 | 0 | |
| RNF157 | 0.3261091 | 0 | |
| CST6 | 0.3259522 | 0 | |
| SLC16A6 | 0.3251537 | 0 | TNF |
| MYO9A | 0.3249451 | 0 | |
| GPR126 | 0.3240376 | 0 | |
| KLHDC7A | 0.3235054 | 0 | |
| KLHL24 | 0.3233948 | 0 | |
| SPATA9 | 0.3219181 | 0 | |
| IL7 | 0.3207665 | 0 | IFN Gamma; IL6/JAK/STAT3 |
| GDPD2 | 0.320576 | 0 | |
| ISL2 | 0.3199193 | 0 | |
| KLHL32 | 0.3194015 | 0 | |
| POU4F1 | 0.3187364 | 0 | |
| DCP1B | 0.3165805 | 0 | |
| SPRY1 | 0.3163539 | 0 | |
| IL33 | 0.3161666 | 0 | IL2/JAK/STAT5 |
| PODXL | 0.3153704 | 0 | KRAS up |
| DNAJC12 | 0.3149573 | 0 | |
| RECK | 0.3131702 | 0 | |
| RWDD2A | 0.3130202 | 0 | |
| FBXO17 | 0.3128216 | 0 | |
| CCBL2 | 0.3117943 | 0 | |
| LOXL1 | 0.3101832 | 0 | |
| SCAMP5 | 0.3097628 | 0 | EMT |
| SPATA17 | 0.3094796 | 0 | |
| USP13 | 0.3088522 | 0 | |
| CAR13 | 0.307364 | 0 | |
| ANGEL1 | 0.3062707 | 0 | |
| INSL6 | 0.3058079 | 0 | |
| CD200 | 0.3056026 | 0 | |
| EPB41L5 | 0.3028196 | 0 | |
| CUGBP2 | 0.3021463 | 0 | |
| PPM1E | 0.3013568 | 0 | |
| SEMA3E | 0.3001962 | 0 | |
| GPR146 | 0.2996591 | 0 | |

| Gene Symbo | Fold Chang | q-Value% | Hallmark Gene Pathway |
|---|---|---|---|
| TRPV4 | 0.2980656 | 0 | |
| STRBP | 0.2978494 | 0 | |
| RSPO2 | 0.2975823 | 0 | |
| EDN1 | 0.2967688 | 0 | |
| IGSF5 | 0.2963914 | 0 | KRAS down; TNF; Inflammatory response |
| HOXB13 | 0.2958655 | 0 | |
| SLC24A3 | 0.2947378 | 0 | |
| USP11 | 0.294686 | 0 | |
| TXNDC16 | 0.2936046 | 0 | Coagulation |
| SEPP1 | 0.2921447 | 0 | |
| LHFPL4 | 0.2907055 | 0 | KRAS down; Androgen |
| TMEM44 | 0.2904531 | 0 | |
| SETBP1 | 0.2904461 | 0 | |
| COLEC12 | 0.2902561 | 0 | |
| CCDC46 | 0.2890813 | 0 | |
| CFHR2 | 0.2880706 | 0 | KRAS up |
| IMPG2 | 0.2865493 | 0 | |
| NUDT12 | 0.2847481 | 0 | |
| DDAH2 | 0.2838525 | 0 | Xenobiotic metabolism |
| CDH11 | 0.2837274 | 0 | EMT; Apical Junction |
| PARD6B | 0.2831091 | 0 | |
| SAT2 | 0.2823593 | 0 | |
| NEO1 | 0.2820792 | 0 | |
| CD59A | 0.2777286 | 0 | |
| KLHL23 | 0.2776508 | 0 | |
| HCN1 | 0.2763676 | 0 | |
| SLC1A6 | 0.2758144 | 0 | |
| MEIS3 | 0.2716381 | 0 | |
| ANGPT4 | 0.2710504 | 0 | |
| CTSH | 0.2685466 | 0 | Coagulation |
| MTM1 | 0.2684281 | 0 | |
| PLA2G2E | 0.2679425 | 0 | |
| RENBP | 0.2679165 | 0 | |
| MCTP1 | 0.2656657 | 0 | |
| PDZD4 | 0.2628054 | 0 | |
| GALNTL2 | 0.2592854 | 0 | |
| TSPAN2 | 0.2592675 | 0 | |
| GGH | 0.2581617 | 0 | UV |
| YPEL1 | 0.2577562 | 0 | KRAS down |
| SLC7A2 | 0.2570398 | 0 | Inflammatory response response |
| SMARCA1 | 0.2560486 | 0 | |

| Gene Symbol | Fold Change | q-Value% | Hallmark Gene Pathway |
|---|---|---|---|
| FGD4 | 0.2556763 | 0 | |
| FBXL16 | 0.2533563 | 0 | |
| DTNA | 0.253257 | 0 | |
| MARCH11 | 0.2514982 | 0 | |
| CRYL1 | 0.2514266 | 0 | |
| RGL3 | 0.2509139 | 0 | |
| SEMA3F | 0.2505757 | 0 | |
| DMXL2 | 0.2501459 | 0 | |
| DUSP22 | 0.2484219 | 0 | |
| PPFIBP2 | 0.244938 | 0 | |
| OGDHL | 0.2447734 | 0 | |
| GLT1D1 | 0.2416123 | 0 | |
| RALGAPA2 | 0.2412746 | 0 | |
| ZEB2 | 0.2394539 | 0 | |
| RRAGD | 0.2388284 | 0 | Glycolysis; IL2/JAK/STAT5 |
| GNAZ | 0.237029 | 0 | |
| SOX21 | 0.2360614 | 0 | |
| OFD1 | 0.2350178 | 0 | |
| TTLL11 | 0.2343399 | 0 | |
| ZFHX4 | 0.2326535 | 0 | |
| NAP1L3 | 0.2324829 | 0 | |
| TSPAN15 | 0.2318296 | 0 | |
| ME3 | 0.2303501 | 0 | |
| SYT8 | 0.2259467 | 0 | |
| XPO7 | 0.2244531 | 0 | |
| ST8SIA4 | 0.2238467 | 0 | IFN Gamma; Allograft rejection |
| HOXC6 | 0.2216182 | 0 | |
| SPIN2 | 0.2211327 | 0 | |
| GPNMB | 0.2209319 | 0 | KRAS up |
| INSC | 0.2200658 | 0 | |
| MTUS2 | 0.2184725 | 0 | |
| ENPP4 | 0.2167498 | 0 | |
| MLF1 | 0.2165704 | 0 | |
| B3GALT1 | 0.2164873 | 0 | |
| CHKA | 0.2163673 | 0 | UV |
| IFITM10 | 0.2138907 | 0 | |
| CCDC39 | 0.2121923 | 0 | |
| GALNTL4 | 0.2118544 | 0 | |
| CCDC125 | 0.2110675 | 0 | |
| TTC14 | 0.2106666 | 0 | |
| FBXL21 | 0.207789 | 0 | |

| Gene Symb | Fold Chang | q-Value% | Hallmark Gene Pathway |
|---|---|---|---|
| HS6ST2 | 0.2072529 | 0 | Glycolysis |
| SYNGR1 | 0.2064028 | 0 | |
| PRKAA2 | 0.201269 | 0 | |
| PHYHD1 | 0.199382 | 0 | |
| MGP | 0.1956566 | 0 | EMT |
| SCML2 | 0.1954414 | 0 | |
| SIM2 | 0.193023 | 0 | |
| BEND4 | 0.1912804 | 0 | |
| MAP2 | 0.1907314 | 0 | |
| RHPN2 | 0.1855907 | 0 | |
| UNC5C | 0.1829063 | 0 | Hedgehog |
| F11R | 0.1824838 | 0 | |
| AK3 | 0.1812055 | 0 | Glycolysis |
| FAM65C | 0.1805073 | 0 | |
| SNRPN | 0.1788775 | 0 | |
| PELI2 | 0.176793 | 0 | |
| PLCG2 | 0.1746671 | 0 | |
| KRBA1 | 0.1745453 | 0 | |
| SNURF | 0.174417 | 0 | |
| RANBP17 | 0.1720534 | 0 | |
| ZFP354B | 0.169886 | 0 | |
| RFTN1 | 0.1666373 | 0 | |
| BCHE | 0.1654202 | 0 | |
| ACTR3B | 0.1643384 | 0 | |
| NUP210 | 0.1624332 | 0 | |
| GDPD1 | 0.159657 | 0 | |
| RAB7L1 | 0.1593422 | 0 | |
| PHACTR2 | 0.1578884 | 0 | |
| HS3ST1 | 0.1542789 | 0 | |
| TPH2 | 0.1514165 | 0 | |
| AOAH | 0.1486633 | 0 | |
| ARRDC4 | 0.1469872 | 0 | |
| UNC13C | 0.1458555 | 0 | |
| CPEB1 | 0.1453479 | 0 | |
| TNFRSF11B | 0.1446051 | 0 | EMT; Apical Junction |
| NPY1R | 0.143653 | 0 | |
| LRFN3 | 0.1423139 | 0 | |
| IL6 | 0.1412925 | 0 | Inflammatory response; IFN Gamma; TNF; E |
| MFSD6 | 0.1394191 | 0 | KRAS down |
| ACOX2 | 0.1339345 | 0 | Xenobiotic metabolism; Coagulation |
| HOXC9 | 0.1324764 | 0 | |

| Gene Symb | Fold Chang | q-Value% | Hallmark Gene Pathway |
|---|---|---|---|
| TIFA | 0.1286579 | 0 | |
| HOXC13 | 0.1270609 | 0 | |
| PFN4 | 0.1260621 | 0 | |
| PCDHB13 | 0.1255327 | 0 | |
| PMAIP1 | 0.1243457 | 0 | Apoptosis |
| AKIP1 | 0.1237161 | 0 | |
| ZFP667 | 0.1205631 | 0 | |
| GAS6 | 0.1170317 | 0 | |
| CHST10 | 0.1147786 | 0 | |
| ARHGAP44 | 0.1147587 | 0 | |
| SNED1 | 0.1123648 | 0 | |
| PCTP | 0.1116766 | 0 | |
| ACOT1 | 0.1069074 | 0 | |
| EPB41L3 | 0.1060608 | 0 | KRAS up |
| CFH | 0.1053464 | 0 | KRAS up; Coagulation; IFN Gamma |
| STAU2 | 0.1053276 | 0 | |
| CFHR1 | 0.1050475 | 0 | |
| CPA6 | 0.1026295 | 0 | |
| PITX2 | 0.0988533 | 0 | |
| PRR5L | 0.0965385 | 0 | |
| RNF32 | 0.0917558 | 0 | |
| PGAP1 | 0.0912664 | 0 | |
| NOX4 | 0.0903302 | 0 | |
| PYGO1 | 0.0885216 | 0 | |
| SGK3 | 0.08813 | 0 | |
| LCN2 | 0.087105 | 0 | |
| FAM151B | 0.0847959 | 0 | |
| LRBA | 0.0847579 | 0 | |
| REX2 | 0.0833364 | 0 | |
| INADL | 0.0829624 | 0 | |
| SCARA3 | 0.0806106 | 0 | |
| KIF6 | 0.0796968 | 0 | |
| MAPK4 | 0.07841 | 0 | |
| SCN5A | 0.0742769 | 0 | |
| NME5 | 0.0698953 | 0 | |
| MGST1 | 0.0693365 | 0 | |
| POSTN | 0.068332 | 0 | EMT; Angiogenesis |
| WISP2 | 0.0670486 | 0 | |
| CCBE1 | 0.0624573 | 0 | |
| PERP | 0.0605548 | 0 | p53 |
| SLC35F1 | 0.0599989 | 0 | |

| Gene Symbo | Fold Chang | q-Value% | Hallmark Gene Pathway |
|---|---|---|---|
| HEPH | 0.0590415 | 0 | |
| TOM1L1 | 0.0578504 | 0 | |
| PNMA2 | 0.0548619 | 0 | |
| GCA | 0.0544092 | 0 | |
| CHMP4C | 0.0523765 | 0 | |
| PRRX1 | 0.0502006 | 0 | KRAS up; EMT |
| BDH2 | 0.0481637 | 0 | Oxidative Phosphorylation |
| NKAIN2 | 0.0446705 | 0 | |
| PRR16 | 0.0446324 | 0 | |
| EIF2C4 | 0.0436899 | 0 | |
| ATF7IP2 | 0.0414438 | 0 | |
| JPH1 | 0.0407973 | 0 | |
| AQP5 | 0.0404256 | 0 | |
| MEGF10 | 0.0400179 | 0 | |
| SORBS2 | 0.0390835 | 0 | |
| PKIA | 0.037122 | 0 | |
| EPHA7 | 0.035353 | 0 | |
| ZFP521 | 0.0338215 | 0 | |
| PLCH1 | 0.0307651 | 0 | |
| ZFP2 | 0.0301177 | 0 | |
| TSPAN12 | 0.0297926 | 0 | |
| PCDH9 | 0.0204579 | 0 | |
| HOXD8 | 0.0196795 | 0 | |
| RRAGB | 0.0173568 | 0 | |
| GSPT2 | 0.0160708 | 0 | |
| | | | |
| | | | |
| | | | |
| | | | |
| | | | |
| | | | |

**Genes overlapping in Oncomine Datasets**

| **Gene Symbol** | |
|---|---|
| ANKS1B | 2.998855 |
| ARHGAP8 | 2.8466609 |
| ARRDC4 | 2.3240871 |
| ASPN | 0.2202643 |
| BCAT1 | 3.0917392 |
| BLNK | 1.5792677 |
| C11orf88 | 0.6578947 |
| C13orf15 | 0.8333333 |
| CACNB2 | 0.204918 |
| CALCRL | 0.3546099 |
| CD248 | 0.286533 |
| CD80 | 0.7518797 |
| CSGALNACT | 0.5319149 |
| CTGF | 0.4424779 |
| DPEP1 | 0.6993007 |
| DTNA | 1.5185031 |
| EIF2C4 | 1.3498257 |
| ERGIC1 | 1.4231067 |
| ESYT2 | 2.9073348 |
| F2R | 0.3745318 |
| FAM65C | 1.4913656 |
| FBLN1 | 0.5102041 |
| FLRT3 | 6.378023 |
| GALNTL2 | 0.1824818 |
| GCA | 3.8580275 |
| GCNT1 | 3.8320305 |
| HOXB13 | 0.245942 |
| HS3ST3A1 | 4.7002573 |
| HSD17B1 | 0.5681818 |
| HSPB1 | 1.5600429 |
| IGFBP6 | 0.1273885 |
| IL6 | 0.6666667 |
| ITGB3 | 0.4784689 |
| JPH2 | 0.3546099 |
| KLK6 | 0.0723589 |
| LONRF3 | 0.6410256 |
| LRBA | 1.4777253 |
| MAF | 0.5524862 |
| MAP2 | 2.102491 |
| | |

| **Gene Symbol** | |
|---|---|
| MCTP1 | 0.5649718 |
| MEGF10 | 0.4273504 |
| MMP2 | 0.3134796 |
| MURC | 2.226442 |
| NKAIN2 | 0.6329114 |
| OMD | 0.3424658 |
| PLA2G2E | 1.1600676 |
| POP1 | 0.6802721 |
| RALGAPA2 | 0.2 |
| RALGPS2 | 1.2887355 |
| RGNEF | 2.4473855 |
| RNF157 | 2.3123338 |
| RPS6KA2 | 0.4201681 |
| RRAD | 0.6493506 |
| SEC16B | 1.4772748 |
| SEMA4D | 2.3452744 |
| SH3GL3 | 1.5667957 |
| SIGIRR | 1.7877226 |
| SLC9A2 | 0.6711409 |
| SMG6 | 1.8959751 |
| SNRPN | 1.5784025 |
| SNURF | 1.1707317 |
| SOX11 | 0.106383 |
| ST8SIA4 | 2.1841192 |
| STAC2 | 0.4201681 |
| SYNGR1 | 1.5265292 |
| TAF9B | 1.2718738 |
| TBC1D9 | 2.1934848 |
| TRAPPC9 | 1.5082319 |
| TRIM66 | 0.1862197 |
| TSPAN12 | 0.877193 |
| ZFHX4 | 0.1322751 |

**Metabolic Genes and Associated Pathways**

| Oncomine_( | Pathway | Gene_ID |
|---|---|---|
| | KRAS up | ADAM8 a disintegrin and metallopeptidase domain 8(Adam8) Mus musculus |
| | Xeno | ADH7 alcohol dehydrogenase 7 (class IV), mu or sigma polypeptide(Adh7) |
| | Xeno/Adipoge | ALDH2 aldehyde dehydrogenase 2, mitochondrial(Aldh2) Mus musculus |
| | Adipogenesis | ANGPT1 angiopoietin 1(Angpt1) Mus musculus |
| | Glycolysis/KR. | ANGPTL4 angiopoietin-like 4(Angptl4) Mus musculus |
| | Glycolysis | ARTN artemin(Artn) Mus musculus |
| | Glycolysis | B3GNT3 UDP-GlcNAc:betaGal beta-1,3-N-acetylglucosaminyltransferase 3(B3gnt3) |
| Yes | mTORC/Xeno | BCAT1 branched chain aminotransferase 1, cytosolic(Bcat1) Mus musculus |
| | KRAS up | BTC betacellulin, epidermal growth factor family member(Btc) Mus musculus |
| | KRAS down | CAMK1D calcium/calmodulin-dependent protein kinase ID(Camk1d) Mus musculus |
| Yes | KRAS down | CD80 CD80 antigen(Cd80) Mus musculus |
| | Glycolysis/KR. | CHST2 carbohydrate sulfotransferase 2(Chst2) Mus musculus |
| | Peroxisome | CRABP2 cellular retinoic acid binding protein II(Crabp2) Mus musculus |
| | KRAS down | EDN2 endothelin 2(Edn2) Mus musculus |
| | Adipogenesis/ | EPHX2 epoxide hydrolase 2, cytoplasmic(Ephx2) Mus musculus |
| Yes | p53 | F2R coagulation factor II (thrombin) receptor(F2r) Mus musculus |
| Yes | Xeno | FBLN1 fibulin 1(Fbln1) Mus musculus |
| | Peroxisome | GSTK1 glutathione S-transferase kappa 1(Gstk1) Mus musculus |
| | p53 | HIST1H1C histone cluster 1, H1c(Hist1h1c) Mus musculus |
| Yes | Peroxisome | HSD17B11 hydroxysteroid (17-beta) dehydrogenase 11(Hsd17b11) |
| | KRAS up/Xen | ID2 inhibitor of DNA binding 2(Id2) Mus musculus |
| | Xeno | IL1R1 interleukin 1 receptor, type I(Il1r1) Mus musculus |
| | KRAS up | IL2RG interleukin 2 receptor, gamma chain(Il2rg) Mus musculus |
| | KRAS up/Xen | IRF8 interferon regulatory factor 8(Irf8) Mus musculus |
| | KRAS up | ITGBL1 integrin, beta-like 1(Itgbl1) Mus musculus |
| | p53 | JAG2 jagged 2(Jag2) Mus musculus |
| | KRAS up | LAT2 linker for activation of T cells family, member 2(Lat2) Mus musculus |
| | Glycolysis | LHPP phospholysine phosphohistidine inorganic pyrophosphate phosphatase(Lhpp) |
| | Glycolysis | LHX9 LIM homeobox protein 9(Lhx9) Mus musculus |
| | Adipogenesis | LTC4S leukotriene C4 synthase(Ltc4s) Mus musculus |
| | KRAS up | MMD monocyte to macrophage differentiation-associated(Mmd) Mus musculus |
| | KRAS up | MYCN v-myc avian myelocytomatosis viral related oncogene, neuroblastoma derived(Mycn) |
| | Glycolysis | NOL3 nucleolar protein 3 (apoptosis repressor with CARD domain)(Nol3) Mus musculus |
| | Xeno | NQO1 NAD(P)H dehydrogenase, quinone 1(Nqo1) Mus musculus |
| | Glycolysis | NT5E 5' nucleotidase, ecto(Nt5e) Mus musculus |
| Yes | Adipogenesis | OMD osteomodulin(Omd) Mus musculus |
| | OxPhos/Adipo | PHYH phytanoyl-CoA hydroxylase(Phyh) Mus musculus |
| | p53 | PITPNC1 phosphatidylinositol transfer protein, cytoplasmic 1(Pitpnc1) Mus musculus |
| | KRAS up | PLAT plasminogen activator, tissue(Plat) Mus musculus |
| | KRAS up | PLAU plasminogen activator, urokinase(Plau) Mus musculus |
| | KRAS up | PLEK2 pleckstrin 2(Plek2) Mus musculus |
| | KRAS up | PPBP pro-platelet basic protein(Ppbp) Mus musculus |
| | p53 | PROCR protein C receptor, endothelial(Procr) Mus musculus |
| | | |

| Oncomine_O | Pathway | Gene_ID |
|---|---|---|
| | KRAS down | PRODH proline dehydrogenase(Prodh) Mus musculus |
| | Xeno | PTGES prostaglandin E synthase(Ptges) Mus musculus |
| | p53 | PTPRE protein tyrosine phosphatase, receptor type, E(Ptpre) Mus musculus |
| | p53 | PVT1 plasmacytoma variant translocation 1(Pvt1) Mus musculus |
| | Adipogenesis | REEP6 receptor accessory protein 6(Reep6) Mus musculus |
| | p53/KRAS up | RGS16 regulator of G-protein signaling 16(Rgs16) Mus musculus |
| Yes | p53 | RRAD Ras-related associated with diabetes(Rrad) Mus musculus |
| Yes | KRAS up | SEMA3B sema domain, immunoglobulin domain (Ig), short basic domain, secreted, (semaphor |
| | mTORC/Adipo | SLC27A1 solute carrier family 27 (fatty acid transporter), member 1(Slc27a1) Mus musculus |
| | p53 | SLC7A11 solute carrier family 7 (cationic amino acid transporter, y+ system), member 11(Slc7a |
| | Adipogenesis | SNCG synuclein, gamma(Sncg) Mus musculus |
| | Adipogenesis | SORBS1 sorbin and SH3 domain containing 1(Sorbs1) Mus musculus |
| | KRAS up | TFPI tissue factor pathway inhibitor(Tfpi) Mus musculus |
| | Glycolysis | TGFBI transforming growth factor, beta induced(Tgfbi) Mus musculus |
| | KRAS up | TRAF1 TNF receptor-associated factor 1(Traf1) Mus musculus |
| | Glycolysis | VCAN versican(Vcan) Mus musculus |
