# Supplementary material for: Metabolic reprogramming underlies metastatic potential in an obesity-responsive murine model of metastatic triple negative breast cancer
Source: NPJ Breast Cancer. 2017 Jul 17;3:26. doi: 10.1038/s41523-017-0027-5 (PMC5514148; doi:10.1038/s41523-017-0027-5)
Supplement: Supplementary file 6 — Supplemental File 3 [file 41523_2017_27_MOESM6_ESM.pdf]

Probe	p.value	Gene Symbol	Associated Pathway
A_52_P597	1.93E-06	EG70793	
A_52_P101	6.21E-05	6430584L05	
A_51_P385	6.57E-05	TNF	TNF; Inflammatory
A_51_P215	7.40E-05	CACNA1D	
A_52_P161	8.51E-05	LOC620760	
A_52_P135	9.81E-05	LOC673902	
A_51_P386	0.0001238	MORN4	
A_51_P164	0.0001341	SH3KBP1	
A_52_P437	0.0001427	GRIP1	
A_52_P287	0.0001515	STK32C	
A_52_P650	0.0001549	MRPS5	
A_51_P289	0.0001552	AFF3	
A_52_P425	0.0001685	LATS2	
A_51_P335	0.0001902	CHN1	
A_51_P368	0.0001984	DNAHC1	
A_52_P118	0.0002421	9630013D21RIK	
A_51_P491	0.0003361	NFE2L1	
A_51_P125	0.0003729	ACAA2	Fatty acid metabolism
A_51_P292	0.0003914	AUTS2	
A_52_P306	0.0003985	LNX2	
A_51_P235	0.0004308	ZC3H11A	
A_51_P172	0.0004795	5730419I09RIK	
A_51_P188	0.0004894	WDR67	
A_52_P456	0.0005831	4833411C07RIK	
A_51_P203	0.0005866	OLFR1053	Olfactory Transduction
A_51_P218	0.0005919	NDUFA4	Oxidative phosphorylation
A_52_P997	0.0006148	MIER3	
A_52_P590	0.0006274	NBEA	
A_52_P571	0.0006308	B230112C05RIK	
A_52_P517	0.0006322	KLHL23	
A_52_P646	0.0006416	GRPR	
A_51_P161	0.0006991	GM438	
A_52_P550	0.0007279	KCNJ16	
A_52_P630	0.0007286	ULK1	Autophagy
A_52_P340	0.0007717	GNG3	KRAS
A_52_P575	0.0007894	TTC5	
A_52_P382	0.0008159	LOC100044513	
A_52_P497	0.0008364	F830004M19RIK	
A_52_P636	0.000872	PGLYRP2	
A_51_P157	0.0008759	HRASLS5	
A_51_P245	0.0008795	DDIT4	mTOR

Probe	p.value	Gene Symbol	Associated Pathway
A_51_P262	0.0008836	4833421G17RIK	
A_52_P649	0.0009083	PDLIM5	
A_52_P156	0.0009211	POLR3B	
A_52_P118	0.0009377	F830005K03RIK	
A_51_P302	0.0009466	SPN	
A_52_P133	0.000967	OLFR197	Olfactory Transduction
A_52_P136	0.0009939	EG668061	
A_52_P109	0.0003141		
VIRS_1000	2.13E-05		
VIRS_2000	0.0001177		
VIRS_1000	0.0005777		
A_52_P100	0.000124		
VIRS_2000	0.0004385		
VIRS_2000	0.0004277		
VIRS_1000	0.0004432		
VIRS_2000	0.0001014		
VIRS_1000	0.0002979		
VIRS_2000	8.14E-06		
VIRS_1000	0.0008966		
VIRS_1000	0.0001157		
VIRS_1000	8.78E-06		
VIRS_1000	0.0004649		
A_52_P987	0.0004169		
VIRS_1000	0.0001191		
VIRS_1000	0.0005949		
VIRS_1000	0.0005668		
VIRS_1000	9.63E-05		
VIRS_1000	0.000405		
VIRS_2000	0.0005059		
